# Extreme fire weather in Chile driven by climate change and El Niño–Southern Oscillation (ENSO)

**DOI:** 10.1038/s41598-024-52481-x

**Published:** 2024-01-23

**Authors:** Raúl R. Cordero, Sarah Feron, Alessandro Damiani, Jorge Carrasco, Cyrus Karas, Chenghao Wang, Clarisse T. Kraamwinkel, Anne Beaulieu

**Affiliations:** 1 Universidad de Santiago de Chile, Av. Bernardo O’Higgins 3363, Santiago, Chile; 2 Knowledge Infrastructure, University of Groningen, Wirdumerdijk 34, 8911 CE Leeuwarden, The Netherlands; 3 Center for Climate Change Adaptation, National Institute for Environmental Studies, Tsukuba, 305-8506 Japan; 4 University of Magallanes, Av. Manuel Bulnes 1855, 621-0427 Punta Arenas, Chile; 5 School of Meteorology, University of Oklahoma, Norman, OK 73072 USA; 6 Department of Geography and Environmental Sustainability, University of Oklahoma, Norman, OK 73019 USA

**Keywords:** Atmospheric science, Climate change, Natural hazards

## Abstract

A string of fierce fires broke out in Chile in the austral summer 2023, just six years after the record-breaking 2017 fire season. Favored by extreme weather conditions, fire activity has dramatically risen in recent years in this Andean country. A total of 1.7 million ha. burned during the last decade, tripling figures of the prior decade. Six of the seven most destructive fire seasons on record occurred since 2014. Here, we analyze the progression during the last two decades of the weather conditions associated with increased fire risk in Central Chile (30°–39° S). Fire weather conditions (including high temperatures, low humidity, dryness, and strong winds) increase the potential for wildfires, once ignited, to rapidly spread. We show that the concurrence of El Niño and climate-fueled droughts and heatwaves boost the local fire risk and have decisively contributed to the intense fire activity recently seen in Central Chile. Our results also suggest that the tropical eastern Pacific Ocean variability modulates the seasonal fire weather in the country, driving in turn the interannual fire activity. The signature of the warm anomalies in the Niño 1 + 2 region (0°–10° S, 90° W–80° W) is apparent on the burned area records seen in Central Chile in 2017 and 2023.

## Introduction

Although fire activity is determined by several factors including available fuel, land management, and ignition sources^1^, weather is the largest driver of the regional burned area^2,3^. “Fire weather” conditions, including high temperatures, low humidity, dryness, and strong winds, boost wildfire risk and increase the potential for fires, once ignited, to intensify rapidly and spread faster^4^.

Climate change is playing an increasing role in determining wildfire regimes by favoring extreme fire weather conditions^4^. Models suggest that the influence of climate change on fire weather has already emerged in about 22% of global burnable land area^5^. Observations have shown that the global burnable area affected by long fire weather seasons has doubled in recent decades^6^. Hot and dry conditions resulting from rising global temperatures have led to increases in the frequency and severity of fire weather^7^. California^8–10^, southeastern Australia^11^, and Southern Europe^12,13^ have recently seen major wildfire outbreaks associated with extreme fire weather.

Fire activity has dramatically increased in Chile in recent years^14,15^. Fire season in this Andean country peaks in January and February at the height of the southern hemisphere’s summer^16,17^. Around 6000 fires are recorded every year^18^ and a total of 1.7 million ha. burned during the last decade in the country^19^. Fires have serious environmental, economic, and social impacts on their own, but they had devastating effects in recent years as they occurred in combination with a severe drought. Chile is in the midst of a megadrought, the longest in at least 1000 years, that has lowered reservoirs and caused tensions and social unrest over water^20^.

Climate extremes are likely boosting fire activity in Central Chile (30°–39° S). The loss of precipitations observed during the last four decades^21,22^ has been worsened by intense heatwaves^23,24^, which have favored large and high-intensity wildfires in Central Chile. Over 90% of the wildfires in the country occur in this zone, which exhibits a pronounced north–south gradient in precipitation (Fig. S1 and Table S1) and encompasses eight Chilean administrative "Regions". The four southern most of these Chilean Regions (Maule, Biobío, Ñuble, and Araucanía) account for most of the tracts of forest land in the country^18^. Recurrent fires in these densely populated regions have affected crops, led to food shortages for livestock, impacted the economy, and enveloped towns and cities in toxic haze and smoke^17^.

Large-scale natural climate modes, such as El Niño–Southern Oscillation (ENSO), also play a major role in the fire weather and wildfire activity. Although ENSO has large effects on the interannual variability of biomass burning across continents^25^, the footprint of ENSO in the year-to-year changes of the burned area in Chile remains obscured. The phases of ENSO (namely El Niño and La Niña) are driven by the strength of trade winds blowing west along the equator and pushing warm water from South America toward Asia^26^. During El Niño events, trade winds weaken and warm water accumulates nearby the west coast of the Americas. During La Niña events, trade winds strengthen, pushing more warm water toward Asia, increasing upwelling and bringing cold, nutrient-rich water to the surface^26^. Through atmospheric teleconnections, El Niño and La Niña influence the weather worldwide. For example, El Niño makes summer generally warmer in central Chile^27^, which in turn boosts wildfire risk.

In February 2023, the concurrence of a severe drought and persistent heatwaves resulted in extreme fire weather conditions in Central Chile. Just six years after the record-breaking 2017 season, fierce fires raged across the country killing dozens, injuring thousands, and leaving many people homeless. In a matter of weeks blazes obliterated more than 420,000 ha.^19^ making it the country’s second-most destructive fire season on record after 2017^28–30^. The emergency affected many farms as well as large tracts of forest land in three Chilean administrative Regions: Biobío, Ñuble and Araucanía. Copernicus Atmosphere Monitoring Services estimates that during the last fire season alone the Chilean fires released about 4 million tons of carbon into the atmosphere^31^. Early estimates suggest that losses and damages from the last fire season amount to more than 880 million dollars, equivalent to 0.3% of the nation’s current gross domestic product^32^.

In this paper, our main goal is to show how the concurrence of El Niño and climate-fueled droughts and heatwaves has increased the fire risk in Central Chile, thereby contributing the intense fire activity recently seen in the country. Below we analyze the progression of the fire weather in Central Chile (30°–39° S) during the last two decades. As metrics, we use temperature and precipitation data, as well as the dimensionless Fire Weather Index (FWI). The latter is a measure of the fire risk derived solely from weather data^33^, which we took from the atmospheric reanalysis ERA5 produced by the European Centre for Medium-range Weather Forecasts (ECMWF)^34^.

## Results

### Fire weather in the 2023 season

**Figure 1:**
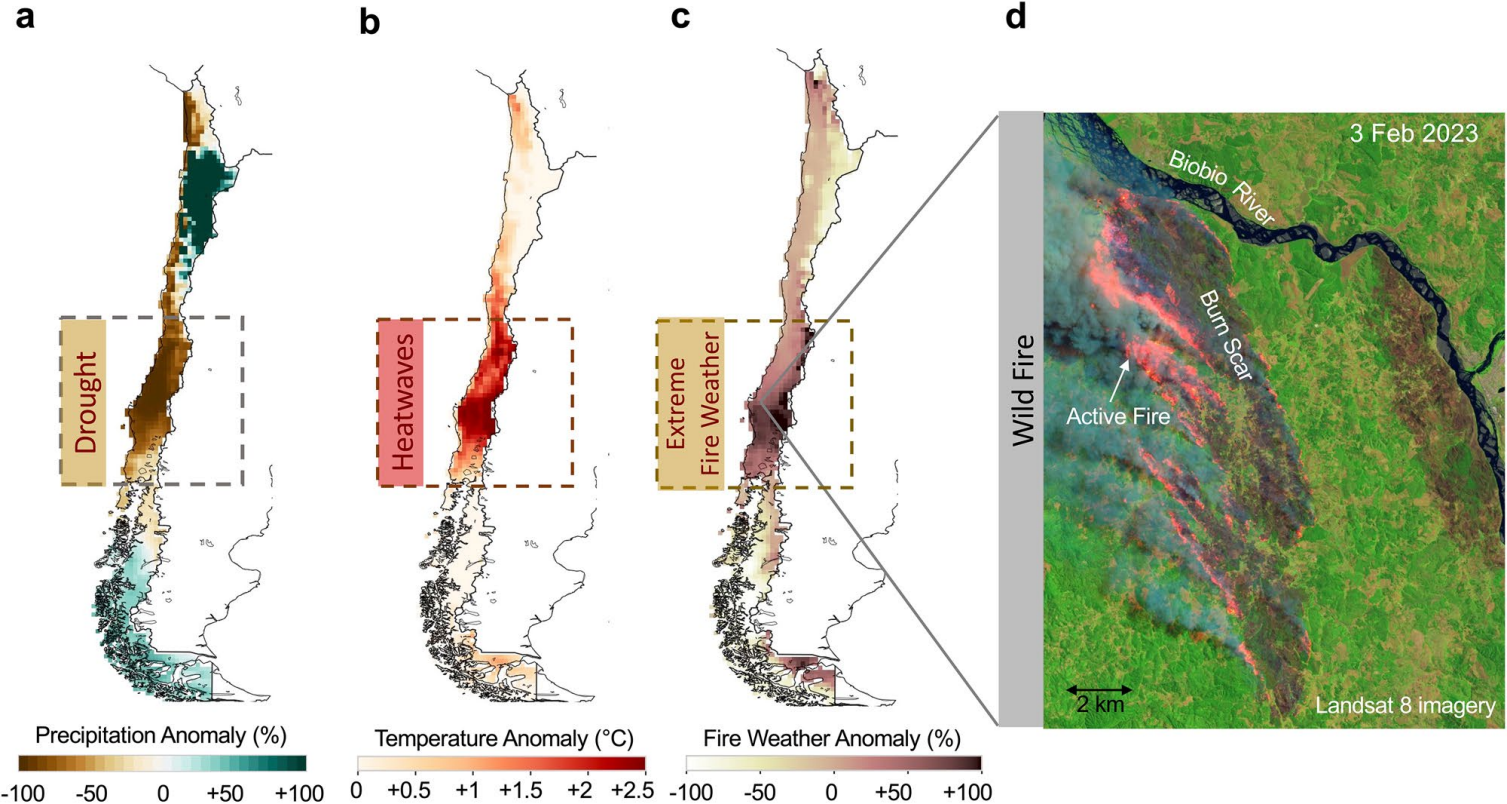
A severe drought worsened by persistent heatwaves fueled fierce fires in February 2023 in Central Chile. (**a**) Precipitation for February 2023 relative to the 1981–2010 mean. The dry February 2023 came on top of the megadrought that has affected central Chile since 2008. (**b**) Air temperature for February 2023 relative to the 1981–2010 mean. February 2023 was the warmest on record in Central Chile. (**c**) Fire Weather Index (FWI) for February 2023 relative to the 1981–2010 mean. Extreme anomalies were registered in the regions severely affected by fires. (**d**) False-color image acquired on 3 February 2023 by the Operational Land Imager (OLI) on Landsat 8 showing the burn scar of Santa Juana Fire, in the BioBio Region, 100 km southeast of Concepcion, the second most populated city in the country. Temperature and precipitation data come from the ERA5 reanalysis^34^. FWI data were computed by using estimates of the precipitations, near-surface wind speed, near-surface temperature, and the relative humidity also from the ERA5 reanalysis. ERA5 data are available at https://www.ecmwf.int/en/forecasts/datasets/reanalysis-datasets/era5. Landsat 8 imagery comes from the NASA Earth Observatory^51^. Plots were generated using Python’s Matplotlib library^52^, version 3.4.3, https://matplotlib.org/3.4.3/contents.html.

A severe drought (with precipitation deficits exceeding 50%; Fig. 1a), exacerbated by persistently high temperatures (up to 2 °C above typical values; Fig. 1b), resulted in exceptionally severe fire weather conditions in Central Chile in February 2023 (with a FWI up to 100% above the long-term average; Fig. 1c). These extreme conditions favored the spread of a series of intense fires, including the Santa Juana Fire (Fig. 1d), one of the largest ever recorded in Chile. This fire alone consumed approximately 75,000 ha. in the BioBio Region, located 100 km southeast of Concepcion, the country's second most populous city.

Extremely dry downslope winds, referred to as *Puelche* wind by locals^35–37^ played an important role in the extreme fire weather conditions observed in Central Chile in February 2023 (Fig. 1c). The complicated topography surrounding cities and towns in Central Chile (Fig. S2) enables *Puelche* winds enable the air to compress, heat up, and dry out as it descends from the Andes into the valleys. Akin to the Santa Ana winds in southern California^37^, *Puelche* winds bring the highes﻿t temperatures and the lowest relative humidities of the year to our study area.

**Figure 2:**
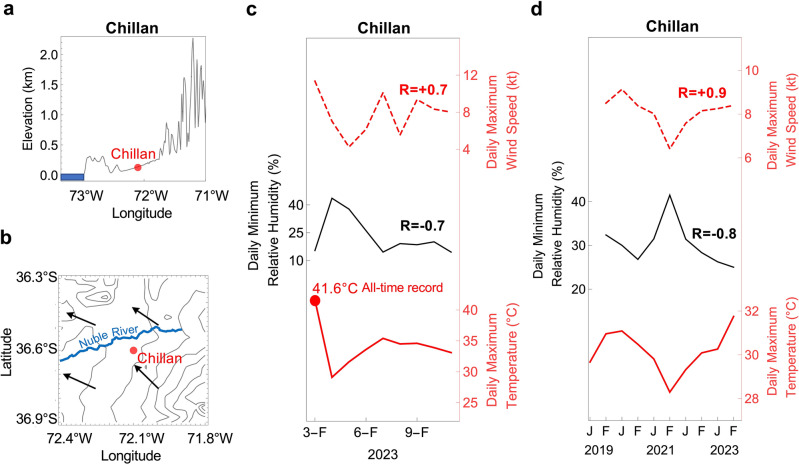
Extreme temperatures in the Chilean valleys are coupled with a low humidity and strengthened winds. (**a**) Elevation along latitude 36° 36′ 24″ S (Chillan is located at 36° 36′ 24″ S, 72° 6′ 12″ W). The topography surrounding cities in the Chilean valleys enables downslope winds. (**b**) Vector array map showing daily maximum (3-h mean) wind direction on the 800-hPa surface on 3 February 2023. Strengthened downslope easterly winds lead to extreme temperatures in Chilean valleys. (**c**) Daily maximum temperature (red line) and the concurrent daily minimum relative humidity (black line) and daily maximum wind speed (dotted red line) measured in Chillan in early February 2023. The city hit its all-time record (41.6 °C) on 3 February 2023. The correlation coefficients (R) between the daily maximum temperature and the relative humidity (as well as between the daily maximum temperature and the wind speed) are shown in the plot. Additional statistics are shown in Table 1. (**d**) Monthly averages of the daily maximum temperature (red line) and the concurrent daily minimum relative humidity (black line) and daily maximum wind speed (dotted red line), measured in Chillan in January and February over the period 2019–2023. There is a strong correlation (R =  + 0.9) between the temperature and the wind speed; there also is a strong anticorrelation (R = − 0.8) between the temperature and the relatively humidity. Additional statistics are shown in Table 1. In plots (**c**) and (**d**), the relative humidity and the wind speed were averaged from 3 to 6 pm local time, period within which the daily maximum temperature generally occurs. The Shuttle Radar Topography Mission (SRTM) 30 m digital elevation model (DEM) provided by USGS (https://earthexplorer.usgs.gov/) was used in (**a**). Wind direction data in plot (**b**) come from the ERA5 reanalysis^34^. Weather measurements (temperature, relative humidity, wind speed and wind direction) come from the Chilean Weather Service (DMC): https://climatologia.meteochile.gob.cl/application/index/menuTematicoEmas. Plots were generated using Python’s Matplotlib library^52^, version 3.4.3, https://matplotlib.org/3.4.3/contents.html.

Powered by *Puelche* winds, on February 3, 2023, the temperature in Chillan (36° 36ʹ S, 72° 6ʹ W), located in the Ñuble Region, reached a daily maximum of 41.6 °C. This local all-time record coincided with extremely dry air due to the prevailing downslope winds (Fig. 2a–c). The influence of *Puelche* winds explains why in Chillan high summer temperatures are strongly correlated (R >  ± 0.8) with dry air and strong winds (Fig. 2d and Table 1). Ground-based measurements conducted by the Dirección Meteorológica de Chile (DMC) show similar strong positive correlations (negative correlations) between temperature and wind speed (between temperature and relative humidity) in other major cities in Central Chile (Fig. S3 and Table 1). *Puelche* winds fanned the flames and hampered efforts to tackle dozens of fires across Central Chile in February 2023.

**Table 1:** Correlation coefficients (R) and p-values between the variables shown in Fig. 2. Correlations considered significant (p < 0.05) are highlighted in bold.

	Maximum temperature (°C)				Minimum relative humidity (%)			
	Daily		Monthly		Daily		Monthly	
	R	p	R	p	R	p	R	p
Minimum relative humidity (%)	**− 0.7**	**0.03**	**− 0.8**	**0.02**				
Maximum wind speed (m/s)	**+ 0.7**	**0.04**	**+ 0.9**	**0.00**	− 0.6	0.07	**− 0.8**	**0.03**

### Latest season seen in context

**Figure 3:**
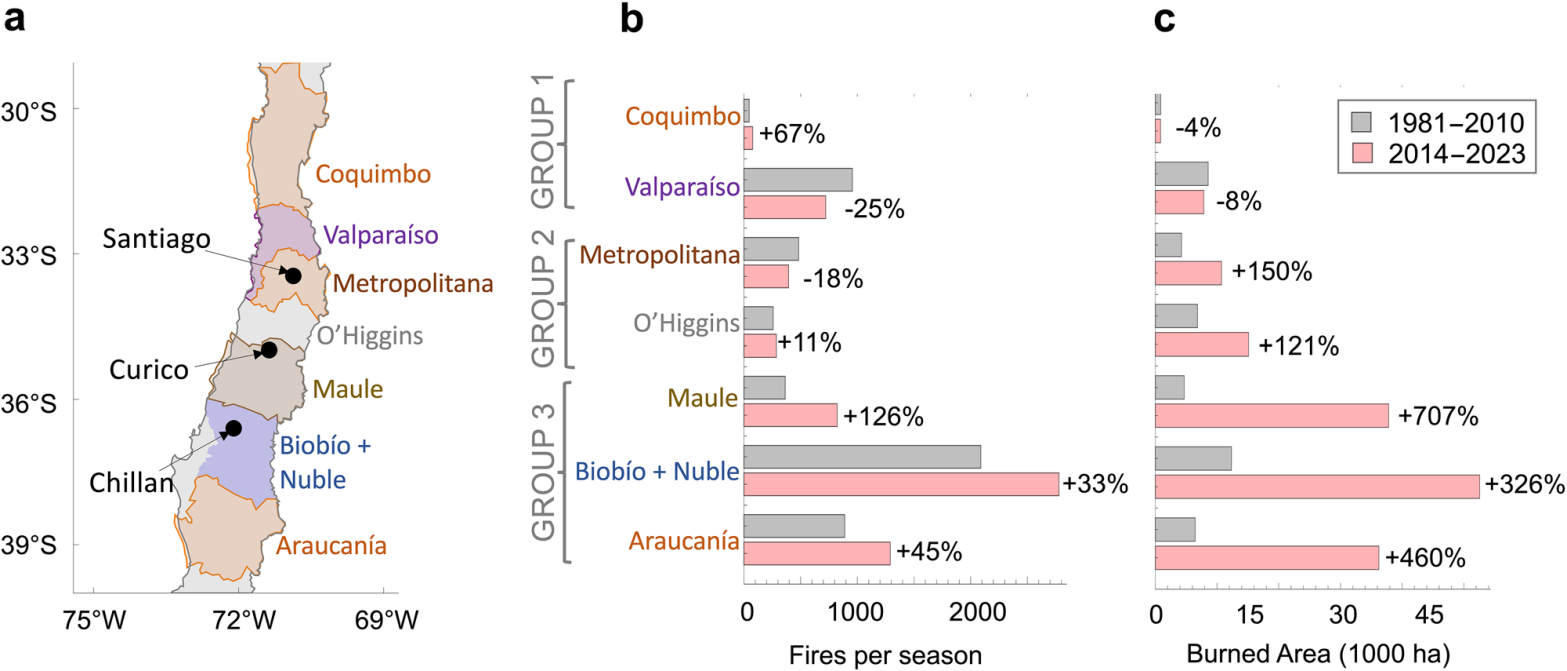
The burned area dramatically increased in Central Chile during the last decade. (**a**) More than 90% of the wildfires in the country occur in Central Chile (30°–39° S) that encompasses eight Chilean administrative Regions: Coquimbo, Valparaiso, Metropolitana, O’Higgins, Maule, Biobío, Ñuble, and Araucanía. (**b**) Change from 1981–2010 to 2014–2023 in the number of fires. While the number of fires has slightly dropped in the Valparaiso and Metropolitana regions, considerable escalations have been observed in the other analyzed Chilean Regions. (**c**) Change from 1981–2010 to 2014–2023 in the burned area. Although the annual burned area has slightly decreased in the Coquimbo and Valparaiso regions, sharp increases have been observed in the other analyzed Chilean Regions. Statistical significance tests (Tables S2, S3) confirm that the fire metrics (number of fires and burned area) of the southernmost Regions (Group 3) are significantly different from fire metrics in other regional groupings. Fire data come from the Chilean Forestry Agency (CONAF)^19^ available at https://www.conaf.cl/incendios-forestales/incendios-forestales-en-chile/estadisticas-historicas/. Plots were generated using Python’s Matplotlib library^52^, version 3.4.3, https://matplotlib.org/3.4.3/contents.html.

The 2023 season does not stand alone. Both the number of fires and the burned area have climbed in recent years (Fig. 3). Considerable escalations in the number of fires have been observed in six of the eight Chilean administrative Regions within our study area, especially in Maule where the number of fires has doubled in recent decades (Fig. 3b). Although only about 1% of the nearly 6000 fires recorded every year in Chile become “large” fires, they account for about 70% of the annual burned area in the country (Fig. S4). “Large” fire is an arbitrary designation, e.g., the Chilean Forestry Agency (CONAF, by its Spanish acronym) considers it to be 200 ha. or more^19^.

At a national level, the burned area nearly tripled from 1981–2010 to 2014–2023 with sharp escalations in most of Central Chile (Fig. 3c). Ranked by the annual burned area, six of the seven most destructive fire seasons on record occurred during the last decade, and a total of about 1.7 million ha. burned during the last decade in the country. In few years, the annual burned area doubled in the Metropolitana and O’Higgins, quadruplicated in BioBio and Nuble, and quintupled in Araucania. A record increase has been observed in Maule (Fig. 3c). The annual burned area has slightly fallen in the northernmost Chilean Regions in our study area (Coquimbo and Valparaiso).

The fire metrics (number of fires and burned area) in the southernmost Regions (Maule, BioBio, Nuble and Araucania) are significantly different from fire metrics in other regional groupings (Tables S2, S3). These results were expected since these four Chilean Regions account for most of the tracts of forest land in the country and are also the wettest in our study area (Fig. S1).

### Climate-fueled droughts and heatwaves

**Figure 4:**
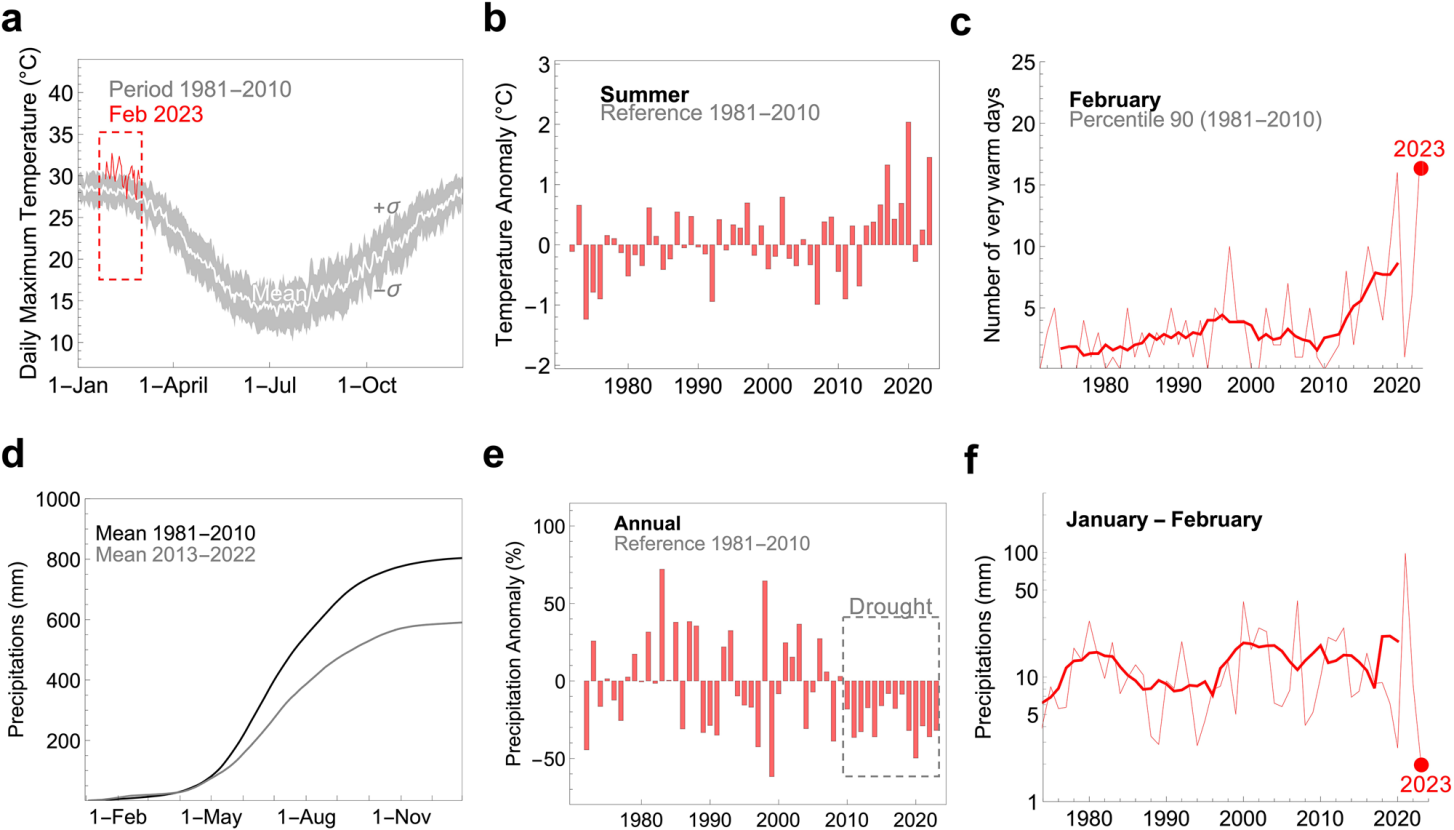
Rising temperatures and persistent droughts have considerably enhanced the fire risk in Central Chile. (**a**) Daily maximum temperature averaged across our study area. For each day of year (DOY), we formed datasets using daily maximum temperatures over the period 1981–2010. The mean (white line) and standard deviation (bounds of the gray shading) of these datasets are shown in the plot. The daily maximum temperature for February 2023 (a period of very warm days) is also shown (red line). (**b**) Summer (December-January–February) temperature relative to the 1981–2010 mean. Five of the ten largest positive anomalies occurred since 2016. (**c**) Progress of ‘very warm” February days in our study area. We consider a day to be “very warm” if the corresponding maximum temperature falls above the 90th percentile of the daily base climatology (built up by using daily maximum temperatures measured over a 30-year base period 1981–2010; see “Methods”). The bold line shows the 7-year centered moving average. A record-breaking number of very warm days was registered in February 2023. (**d**) Accumulated precipitation in our study area averaged over two periods: 1981–2010 (black line) and 2013–2022 (gray line). (**e**) Annual precipitation in our study area relative to the 1981–2010 mean. The dotted rectangular box highlights the megadrought that began in 2008. (**f**) Progress of January–February precipitation in our study area. The bold line shows the 7-year centered moving average. A record-breaking drought was registered in January–February 2023. Temperature and precipitation data come from ERA5 reanalysis^34^ available at https://www.ecmwf.int/en/forecasts/datasets/reanalysis-datasets/era5. Plots were generated using Python’s Matplotlib library^52^, version 3.4.3, https://matplotlib.org/3.4.3/contents.html.

During the exceptionally warm February 2023, the warmest on record in Central Chile, intense heatwaves pushed the daily maximum temperature well above typical conditions (Fig. 4a). This contributed to making the austral summer 2022–2023 the second warmest on record (Fig. 4b). These records are likely part of a broader climate-fueled trend. Five of the ten largest positive temperature anomalies in Central Chile have occurred since 2016 (Fig. 4b), and both heatwaves and “very warm” days have nearly tripled in recent decades (Fig. S5). The record-breaking number of very warm days in February 2023 (Fig. 4c) contributed to extreme fire weather conditions seen in Central Chile the last season.

On top of the recurring deficits in annual precipitation that have affected our study area in recent years (Fig. 4d,e), precipitation in Central Chile reached a new record low in January–February 2023 (Fig. 4f). As is the case in other Mediterranean-like climate zones around the world, dry summer conditions are considered typical in Central Chile. A semi-permanent ridge of high pressure during the summer months keeps Central Chile relatively dry, with the exception of occasional passing frontal systems (and cut-off lows). Yet, total precipitation in Central Chile in January–February 2023 was less than 3 mm, significantly below the 10–20 mm typically recorded in our study area in recent decades (Fig. 4f). The record-breaking drought in January–February 2023 (Fig. 4f) probably favored the last fierce fire season.

The concurrence of severe droughts and persistent heatwaves, that led to the extreme fire weather conditions in Central Chile in February 2023, is becoming more frequent. Five of the ten largest positive FWI anomalies (relative to the 1981–2010 mean) occurred since 2014 (Fig. S6a). Ranked by frequency of extreme fire weather conditions, six of the ten worst seasons occurred since 2014 (Fig. S6b). Extreme fire weather conditions in January 2017 (Fig. S6c) and February 2023 (Fig. S6d) likely contributed to the record fire seasons in 2017 and in 2023. Despite the extreme fire weather conditions in January–February 2020 (Fig. S6b), Chile managed to avoid burned area records in the 2020 season. Few millimeters of well-timed precipitation can make the difference between a relatively mild wildfire season (like in 2022), an above-average but non-record season (like 2020), and a widespread catastrophe (like in 2017 and 2023).

### ENSO teleconnection

The influence of the El Niño and La Niña on the Chilean climate makes the sea surface temperature (SST) of the tropical Pacific of paramount importance. Weekly estimates of the SST in the Niño regions are provided by the Climate Prediction Center (CPC), part of the National Oceanic and Atmospheric Administration (NOAA). Two of the Niño regions currently monitored by NOAA’s CPC are the Niño 1 + 2 Region (right in front of the western coast of Peru; 0°–10° S, 90°–80°W) and the Niño 3.4 Region (in the tropical central Pacific; 5° N–5° S, 170°–120° W). El Niño/La Niña events are classified according to SST anomalies exceeding the ± 0.5 °C threshold in the Niño regions (Fig. S7).

**Figure 5:**
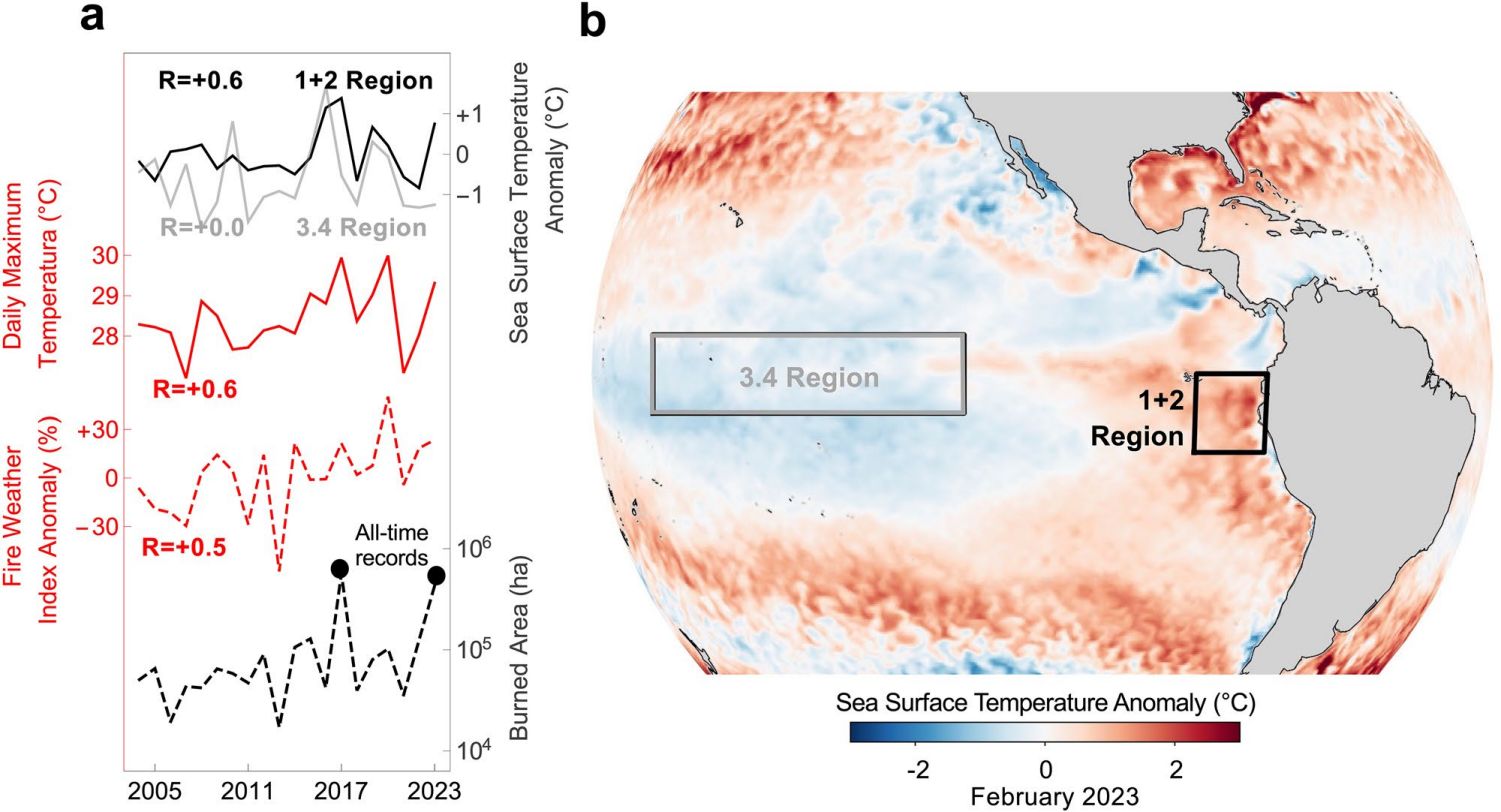
Fire activity in Central Chile is strongly connected with the year-to-year variability in the Niño 1 + 2 region. (**a**) Area annually burned in Central Chile (black dotted line), summer Fire Weather Index (FWI) anomaly averaged across our study area (red dotted line), daily maximum temperature (red line) averaged across Central Chile, and sea surface temperature (SST) anomalies in the Niño 1 + 2 region (black line) and in the Niño 3.4 region (gray line). There is a relatively high correlation between the burned area and the daily maximum temperature (R =  + 0.6) as well as between the burned area and the SST anomaly in the Niño 1 + 2 region (R =  + 0.6). The correlation is slightly lower (R =  + 0.5) between the burned area and the FWI anomaly while there is no correlation (R = 0) between the burned area and SST anomaly in the Niño 3.4 region. Additional statistics are shown in Table 2. Correlation coefficients and p-values per Chilean administrative Region are shown in Table S7. (**b**) The fierce fires in February 2023 in Central Chile concurred with positive SST anomalies in the Niño 1 + 2 region. Contrasting SST anomalies were apparent in February 2023 in the Niño regions. Negative anomalies (La Niña) persisted in the Niño 3.4 region whilst positive anomalies (El Niño) prevailed in the Niño 1 + 2 region. Burned area data come from the Chilean Forestry Agency (CONAF)^19^ available at https://www.conaf.cl/incendios-forestales/incendios-forestales-en-chile/estadisticas-historicas/. SST anomalies in (**a**) (averaged from January to March) are produced by the Climate Prediction Center (CPC), part of the National Oceanic and Atmospheric Administration (NOAA) available at https://www.cpc.ncep.noaa.gov/data/indices/wksst8110.for. Daily maximum temperature (averaged for January–February, at the height of the southern hemisphere’s summer) in (**a**) and SST anomalies (for February 2023) in (**b**) come from the ERA5 reanalysis^34^. FWI data (averaged for the southern hemisphere’s summer) in (**a**) were computed by using estimates of the precipitations, near-surface wind speed, near-surface temperature, and the relative humidity also from the ERA5 reanalysis. ERA5 data are available at https://www.ecmwf.int/en/forecasts/datasets/reanalysis-datasets/era5. Plots were generated using Python’s Matplotlib library^52^, version 3.4.3, https://matplotlib.org/3.4.3/contents.html.

Fire weather conditions in Central Chile are strongly connected with the year-to-year variability of the SST in the Niño 1 + 2 region. El Niño (La Niña) in this region generally leads to warmer (cooler) summers in Central Chile (Fig. S8), subsequently increasing the fire risk. This connection likely explains why the burned area in Central Chile is well correlated (R =  + 0.6) with the SST anomalies in the Niño 1 + 2 region (Fig. 5a and Table 2). Another region of interest, directly off the coast of northern Chile (20°–34° S, 70°–80° W) and referred to as Chile Niño/Niña region, was recently proposed by Xue et al.^38^. The burned area in Central Chile is also correlated (R =  + 0.5) with the SST anomalies in the Chile Niño/Niña region (Fig. S9a), which was expected since the SST in this latter region is firmly coupled with the SST in the Niño 1 + 2 region (Fig. S9b,c).

**Table 2:** Correlation coefficients (R) and p-values between the variables shown in Fig. 5a. Correlations considered significant (p < 0.05) are highlighted in bold.

	Burned area (ha.)		Fire Weather Index (FWI)		Maximum air temperature (°C)		SST ENSO 1 + 2 (°C)	
	R	p	R	p	R	p	R	p
Fire Weather Index (FWI)	**+ 0.5**	**0.04**						
Maximum temperature (°C)	**+ 0.6**	**0.00**	**+ 0.6**	**0.00**				
SST ENSO 1 + 2 (°C)	**+ 0.6**	**0.00**	+ 0.2	0.28	**+ 0.6**	**0.00**		
SST ENSO 3.4 (°C)	+ 0.0	0.83	+ 0.1	0.79	+ 0.3	0.22	**+ 0.5**	**0.01**

The strong connection between the fire weather conditions in Central Chile and the SST in the Niño 1 + 2 region is likely one of the reasons why the fierce fires in February 2023 in Central Chile concurred with positive anomalies in the Niño 1 + 2 region (Fig. 5b). The record fire season in 2017 also coincided with positive anomalies in the Niño 1 + 2 region (Fig. S7). The fire risk in Central Chile appears to be less connected with the interannual variability of the sea surface temperature in the Niño 3.4 region. Temperatures in the Niño 3.4 region and in the Niño 1 + 2 region decoupled in late 2022 and exhibited remarkable contrasting anomalies in late summer 2023 (Fig. S10a). In fact, the record fires in February 2023 in Central Chile concurred with slightly negative anomalies in the Niño 3.4 region (Fig. 5b). The weaker connection between the fire risk in our study area and the Niño 3.4 region explains why we found no correlation (R = 0) between the burned area in Central Chile and SST anomaly in the Niño 3.4 region (Fig. 5a and Table 2).

## Discussion

### Heatwaves and droughts

Climate change is worsening fire weather by making hot and dry conditions more frequent. While the climate-induced signal of excess heat emerged in Central Chile just in the last decade^23,24^, some locations in our study area have been experiencing prolonged drying for nearly 40 years^21,22^. The deteriorating situation in Central Chile has been worsened by a megadrought, which since 2008^20^ has lowered reservoirs and caused tensions over water and may have facilitated larger fires. Ranked by the annual burned area, seven of the ten most destructive fire seasons on record have occurred since 2008 (i.e., the megadrought period).

Precipitation in our study area exhibits a pronounced north–south gradient (Fig. S1), and so do the effects of the megadrought. On average, precipitation in Araucanía (the southernmost Chilean administrative Region considered in this study) is about 8 times greater than precipitation in Coquimbo (the northernmost Chilean administrative Region in this study). Droughts in drier regions tend to suppress fire activity by limiting available fuel, while in wet regions, droughts favor fire spread by drying the already available fuel^4^. This effect may have contributed to the north–south gradient observed in the changes in fire activity shown in Fig. 3c; from 1981–2010 to 2014–2023, the annual burned area slightly decreased in Coquimbo, while it quintupled in Araucanía (Fig. 3c).

Prior efforts^21,22^ suggest that precipitation losses in Central Chile are partially associated with a trend toward the positive phase of the Southern Annular Mode (SAM)^39^. The SAM index describes the strength and position of the Southern Hemisphere westerly winds, which modulates the precipitation regime over the southeast Pacific^40^. The positive SAM trend (Fig. S11a) results from the strengthening and poleward migration of the westerly winds, which in turn has been attributed to both greenhouse gas emissions and the Antarctic ozone depletion^21,22^. The pause (Fig. S11b) in the long-term strengthening of the summer SAM has been credited to the success of the Montreal Protocol that banned human-made ozone-depleting substances^41^. Despite the ozone recovery, a robust positive trend in the SAM is projected under high greenhouse gas emission scenarios^42^. This may result in more droughts for Central Chile in the future.

### El Niño is a risk factor

The strong connection between fire activity in Central Chile and the SST in the Niño 1 + 2 region (Fig. 5a and Table 2) highlights El Niño as an important risk factor. A change of just a few degrees in the tropical Pacific can make the difference between a relatively mild wildfire season and a widespread catastrophe. In fact, the abrupt end of the 2020–2022 triple-dip La Niña (in the Niño 1 + 2 region) likely contributed to the extreme fire weather and the fierce fires that raged across Central Chile in February 2023.

NOAA’s CPC issued its “Final La Niña Advisory” (officially ending the 2020–2022 triple-dip La Niña) in early March 2023^43^. However, four months earlier, in early December 2022, the SST departure from the long-term average in the Niño 1 + 2 region had crossed the − 0.5 °C threshold, effectively ending La Niña (Fig. S10b) in the region that matters the most for Central Chile. Also, in December 2022, Central Chile was hit by an 11-day summer heatwave (the longest ever registered) and experienced temperatures not seen since the beginning of the triple-dip La Niña in late 2020.

CONAF, in charge of coordinating the public efforts aimed at fighting wildfires, may have been caught off guard by the rapid end of the 2020–2022 triple-dip La Niña in the Niño 1 + 2 region (Fig. S10b). La Niña likely contributed to the relatively cool temperatures in central Chile observed around the end of years 2020 and 2021. Summers 2020–2021 and 2021–2022 in central Chile were among the coolest since 2004 and the burned area in the fire season 2021 was the only one below the long-term average seen during the last decade. An extremely warm summer 2022–2023 was considered unlikely by most of the scientists and weather forecasters in Chile in late 2022, when resources and aerial firefighting services were planned. This is why, the Chilean government allocated the last fire season a total of about 95 million dollars for fighting wildfires^44^, a minor increase with respect to the prior season (Fig. S12).

### Other relevant drivers

Despite the paramount importance of El Niño and climate-fueled droughts and heatwaves, other factors also influence fire activity, including the availability of fuel and land management. For instance, plantation forests in the southernmost Chilean Regions considered in this study (Maule, Biobío, Ñuble, and Araucanía) are densely packed with fire-vulnerable trees^45^.

The fire metrics (i.e., the burned area) of plantation forests are not significantly different from the fire metrics of natural forests (Tables S4, S5). Furthermore, regardless of the species, the year-to-year changes in the burned area of natural and plantation forests are both well correlated with SST anomalies in the Niño 1 + 2 region (Table S6). Yet, plantation forests often account for a disproportionate and increasing share of the burned area (Fig. S13a). Although plantations currently cover about 3 million ha., which is roughly 18% of the Chilean forest area and around 4.4% of the total surface of the country^46^, planted forest currently accounts for about 30% of the total burned area (Fig. S13a). This percentage exhibits a considerable interannual variability and nearly doubled in the record seasons 2017 and 2023, which suggest that the predominant planted species (pine and eucalypt, Fig. S13b) fuel large and high-intensity fires under extreme weather conditions.

Plantation forests accounted for about half of the 20% increase (about 3 million ha.) seen in the surface of Chilean forests since the early 1990s^47^. However, the total planted forest area has seen few changes during the last decade (Fig. S13b), a decade marked by the record seasons 2017 and 2023. Following a trend that began in the early 1990s, eucalyptus plantations did considerably increase during the last decade. Mirroring this increment, the relative weight of eucalyptus plantations in the burned planted forest area has nearly doubled since the early 1990s (Fig. S13c). Yet, new tracts of flammable eucalyptus plantations unlikely explain alone the 300% mean surge in the burned area observed in Central Chile from 1981–2010 to 2014–2023.

## Conclusions

Our results suggest that the concurrence of positive SST anomalies in the Niño 1 + 2 region as well as climate-fueled droughts and heatwaves boosts the fire risk in Central Chile. Ranked by frequency of extreme fire weather conditions, six of the ten worst seasons occurred since 2014. Extreme weather (including high temperatures, low humidity, dryness, and strong winds) has decisively contributed to the increased fire activity recently seen in Central Chile.

We have shown that the tropical eastern Pacific Ocean variability modulates the seasonal fire weather in Central Chile, driving in turn the interannual fire activity. The signature of the positive anomalies in the Niño 1 + 2 region is apparent on the burned area records seen in Central Chile in 2017 and 2023. Although the effects of ENSO on the interannual variability of biomass burning across continents is well known^25^, the strong connection between the SST anomalies in the Niño 1 + 2 region and the fire weather conditions in Central Chile was not clear before this study.

Although the funds allocated in Chile for fighting wildfires have doubled over the last decade (Fig. S12), the aftermath of the 2023 fire season suggests that additional investments in adaptation and resilience are necessary. That includes improving the country’s Early Warning System (EWS), a critical tool to take early action, reduce disaster risk, and support climate adaptation. These systems allow forecasting hazardous weather and help to minimize impacts by opportunely informing governments, communities, and individuals. Chile has competent and functional institutions aimed at forecasting and minimizing risks associated with climate-fueled extreme events. However, the fierce fire season 2023 has made the limitations of the Chilean EWS apparent, leaving lessons that authorities must not ignore. The lack of an ENSO forecast tailored to the needs of Chile (and the Niño 1 + 2 region) is probably one of them.

## Methods

### Fire statistics in Chile

Our study area is Central Chile (30°–39° S), which encompasses eight Chilean administrative “Regions” (Fig. 3a). More than 90% of the wildfires in the country occur in these regions. Four of these Chilean Regions (Maule, Biobío, Ñuble, and Araucanía) account for most of the tracts of forest land in the country^18^. We used statistics of fires (Fig. 3) prepared by the Chilean Forestry Agency (CONAF). CONAF is responsible for the management of Chile’s forests and for the prevention, detection and suppression of wildfires in Chile. CONAF data^19^ are available at https://www.conaf.cl/incendios-forestales/incendios-forestales-en-chile/estadisticas-historicas/. Forestry statistics come from the Chilean Office for Agricultural Studies and Policies (ODEPA). ODEPA data^46^ are available at https://www.odepa.gob.cl/estadisticas-del-sector/estadisticas-productivas. Additional forestry figures were also obtained from CONAF^47^ https://www.conaf.cl/nuestros-bosques/bosques-en-chile/catastro-vegetacional/.

### Weather data

Surface weather measurements (temperature, relative humidity, wind speed and wind direction) at specific locations in Central Chile (Fig. 2) come from the Chilean Weather Service (DMC). DMC data are available at https://climatologia.meteochile.gob.cl/application/index/menuTematicoEmas. At locations where surface weather measurements were not available, we used data from the atmospheric reanalysis ERA5 produced by the European Centre for Medium-range Weather Forecasts (ECMWF)^34^. ERA5 data are available at: https://www.ecmwf.int/en/forecasts/datasets/reanalysis-datasets/era5.

### Fire Weather Index (FWI)

In order to analyze the progression of the fire weather in Central Chile (30°–39° S), we used as metrics temperature and precipitation data, as well as the dimensionless Fire Weather Index (FWI) as described by Van Wagner^33^. The FWI is a measure of the fire risk derived solely from weather data (wind speed, rainfall, temperature, and relative humidity) corresponding to the local solar noon (when the sun is at its peak). While the FWI scale is the same everywhere, the level of risk (e.g., very low, low, moderate, etc.) changes with the site or region according to local conditions. Observations of the wind speed, rainfall, temperature, and the relative humidity are sequentially used to compute the Fine Fuel Moisture Code (FFMC), which represents moisture conditions for shaded litter fuels; the Duff Moisture Code (DMCo), which represents fuel moisture of decomposed organic material underneath the litter; the Drought Code (DC), which represents drying deep into the soil; the Initial Spread Index (ISI), which integrates fuel moisture for fine dead fuels and surface windspeed, and represents the spread potential; the Buildup Index (BUI), which combines the current DMCo and DC to produce an estimate of potential heat release in heavier fuels; and finally the Fire Weather Index (FWI), which integrates ISI and BUI.

We computed the FWI by using the XCLIM library^48^. As inputs, we used estimates of the precipitations, near-surface wind speed, near-surface temperature, and the relative humidity from the atmospheric reanalysis ERA5 produced by ECMWF^34^. ERA5 data are available at: https://www.ecmwf.int/en/forecasts/datasets/reanalysis-datasets/era5.

### El Niño Data

In order to analyze the correlation between the burned area and the sea surface temperature (SST) anomalies in the Niño regions, we used data from NOAA’s Climate Prediction Center (CPC) and from the ERA5 dataset produced by ECMWF^34^. NOAA CPC data are available at https://www.cpc.ncep.noaa.gov/data/ indices/wksst8110.for while ERA5 data are available at: https://www.ecmwf.int/en/forecasts/datasets/reanalysis-datasets/era5.

NOAA’s CPC considers that an El Niño episode is characterized by a five consecutive 3-month running mean of SST anomalies in the Niño 3.4 region (5° N–5° S, 170°–120° W), that is above the threshold of + 0.5 °C^26^. According to NOAA’s CPC^26^, the Niño 3.4 region encompasses the western half of the equatorial cold tongue region, which provides a good measure of important changes in the sea surface temperature that affect patterns of deep tropical convection and atmospheric circulation. Three-month running mean of sea surface temperature anomalies in the Niño 3.4 region is used by the NOAA’s CPC for producing the Oceanic Niño Index (ONI), an index widely used by scientists and weather forecasters around the world, including Chile. Monthly NOAA’s CPC assessments are also mostly focused on the Niño 3.4 region. However, climate in Central Chile is strongly influenced by the Niño 1 + 2 region (0°–10° S, 90°–80° W) ^27,40^. In addition to the Niño 1 + 2 and the Niño 3.4, we analyzed a region directly off the coast of northern Chile (20°–34° S, 70°–80° W) referred to as Chile Niño/Niña region^38^.

### Climate extreme analysis

Following prior efforts^49,50^, over a base period of 30 years (1981–2010), we used a 15-day rolling window of the daily estimate of either the daily maximum temperature (TX) or the Fire Weather Index (FWI) in order to form datasets of 450 values for each day. For each day, the dataset mean defined a daily base climatology from which daily anomalies (either for TX or for FWI) were calculated. The histograms of the daily anomalies (the departure of daily estimates from the daily base climatology) enabled us to compute.

- The number of “extreme” fire weather days: the number of days above the 90th percentile of the FWI anomaly distribution corresponding to the base period.
- The number of “very warm” days: the number of days above the 90th percentile of the TX anomaly distribution corresponding to the base period.

Here, we considered a heatwave as a period of at least 3 consecutive “very warm” days.

### Statistical significance tests

Grouping (according to the Chilean administrative Regions and according to the type of forest) was tested by using the Analysis of Variance (ANOVA) and the Mean Difference Test (MDT). In the case of the type of forest (Table S6), we tested the following groups: Grouping 1 = Natural, Grouping 2 = Plantations, Grouping 3 = Other (unclassified) land. In the case of the Chilean administrative Regions (Table S7), we tested the following groups: Grouping 1 = Coquimbo, Valparaíso, Grouping 2 = Metropolitana, O’Higgins, Grouping 3 = Maule, BioBio, Nuble, Araucanía. Fire metrics (number of fires and burned area) registers over the last decade were tested. Here, a p value, or probability value, of 0.05 or less was considered statistically significant. The results of the test are shown in Tables S2–S5. The tests confirmed that, for example, fire metrics (number of fires and burned area) of the southernmost Regions in our study area (Maule, Biobío, Ñuble, and Araucanía) are significantly different from fire metrics in other regional groupings (Tables S2, S3).

### Supplementary Information 

Supplementary Information.

## Data Availability

Fire data come from the Chilean forestry agency (CONAF): https://www.conaf.cl/incendios-forestales/incendios-forestales-en-chile/estadisticas-historicas/. Weather measurements (temperature, relative humidity, wind speed and wind direction) at specific locations in central Chile come from Chilean Weather Service (DMC): https://climatologia.meteochile.gob.cl/application/index/menuTematicoEmas. Weekly sea surface temperature (SST) anomalies in the Niño regions come from NOAA’s Climate Prediction Center (CPC): https://www.cpc.ncep.noaa.gov/data/indices/wksst8110.for. Datasets of the sea surface temperature (SST), precipitations, near-surface wind speed, near-surface temperature, and the relative humidity, come from the atmospheric reanalysis ERA5 produced by the European Centre for Medium-range Weather Forecasts (ECMWF), are available at: https://www.ecmwf.int/en/forecasts/datasets/reanalysis-datasets/era5. Forestry statistics from ODEPA and CONAF are available at https://www.odepa.gob.cl/estadisticas-del-sector/estadisticas-productivas and https://www.conaf.cl/nuestros-bosques/bosques-en-chile/catastro-vegetacional. Images from Landsat 8 Operational Land Imager (OLI) come from the United States Geological Survey (USGS): https://earthexplorer.usgs.gov/.
