# Supplementary material for: Extreme fire weather in Chile driven by climate change and El Niño–Southern Oscillation (ENSO)
Source: Sci Rep. 2024 Jan 23;14:1974. doi: 10.1038/s41598-024-52481-x (PMC10806187; doi:10.1038/s41598-024-52481-x)
Supplement: Supplementary file 1 — Supplementary Information. [file 41598_2024_52481_MOESM1_ESM.pdf]

# Supplementary Information

## Extreme Fire Weather in Chile driven by Climate Change and El Niño–Southern Oscillation (ENSO)

Raúl R. Cordero[1], Sarah Feron[1,2,*], Alessandro Damiani[3], Jorge Carrasco[4], Cyrus Karas[1], Chenghao Wang[5,6], Clarisse T. Kraamwinkel[2], Anne Beaulieu[2]

1 Universidad de Santiago de Chile. Av. Bernardo O'Higgins 3363, Santiago, Chile.
2 Knowledge Infrastructure, University of Groningen, Wirdumerdijk 34, 8911 CE Leeuwarden, Netherlands.
3 Center for Climate Change Adaptation, National Institute for Environmental Studies, Tsukuba 305-8506, Japan
4 University of Magallanes, Av. Manuel Bulnes 1855, 621-0427 Punta Arenas, Chile
5 School of Meteorology, University of Oklahoma, Norman, OK 73072, USA
6 Department of Geography and Environmental Sustainability, University of Oklahoma, Norman, OK 73019, USA

* Corresponding Author
Sarah Feron
s.c.feron@rug.nl

**Precipitations in our study area exhibits a pronounced north-south gradient.**

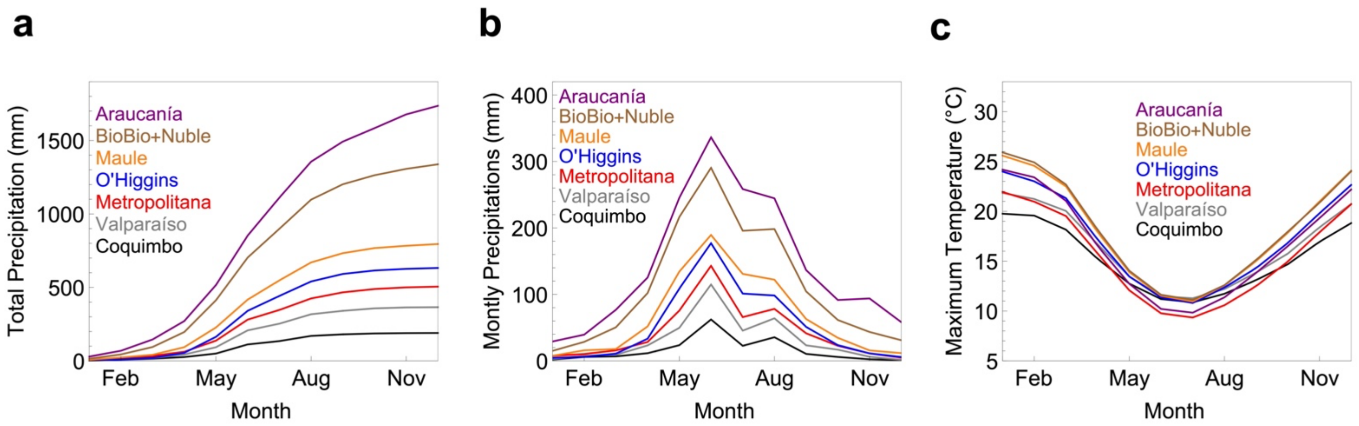


**Fig. S1**

Climatology of the Chilean administrative Regions considered in this study (note that climate conditions can vary within each of the Regions due to their diverse geography and microclimates).

a) Average accumulated precipitation.
b) Average precipitation.
c) Average maximum surface air temperature

Monthly data from Harris, I., *et al.* Version 4 of the CRU TS monthly high-resolution gridded multivariate climate dataset*. Sci. Data* **7**, 109 (2020), over the period 1991-2020, were used. Plots were generated using Python's Matplotlib library, version 3.4.3, https://matplotlib.org/3.4.3/contents.html.

**The topography surrounding cities in Central Chile enables downslope winds.**

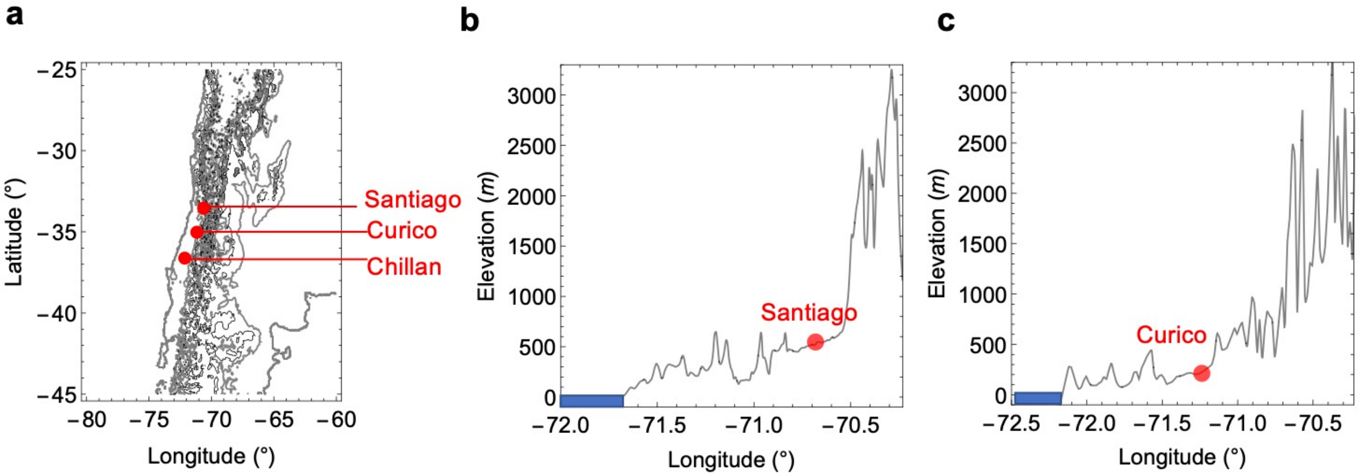


**Fig. S2**

a) Central Chile (30-39°S) is the most populated region in the country. Santiago de Chile is a major mid-latitude Andean city of 7.7 million inhabitants while Curico and Chillan are mid-size cities of less than 200,000 inhabitants.

b) Elevation along latitude 33°27'25"S (Santiago is located at 33°27'25"S, 70°38'54"W).

c) Elevation along latitude 34°58'58"S (Curico is located at 34°58'58"S, 71°14'22"W).

The Shuttle Radar Topography Mission (SRTM) 30 m digital elevation model (DEM) provided by USGS (https://earthexplorer.usgs.gov/) was used. Plots were generated using Python's Matplotlib library, version 3.4.3, https://matplotlib.org/3.4.3/contents.html.

**High temperatures in Central Chile are strongly correlated with dry air and strong winds.**

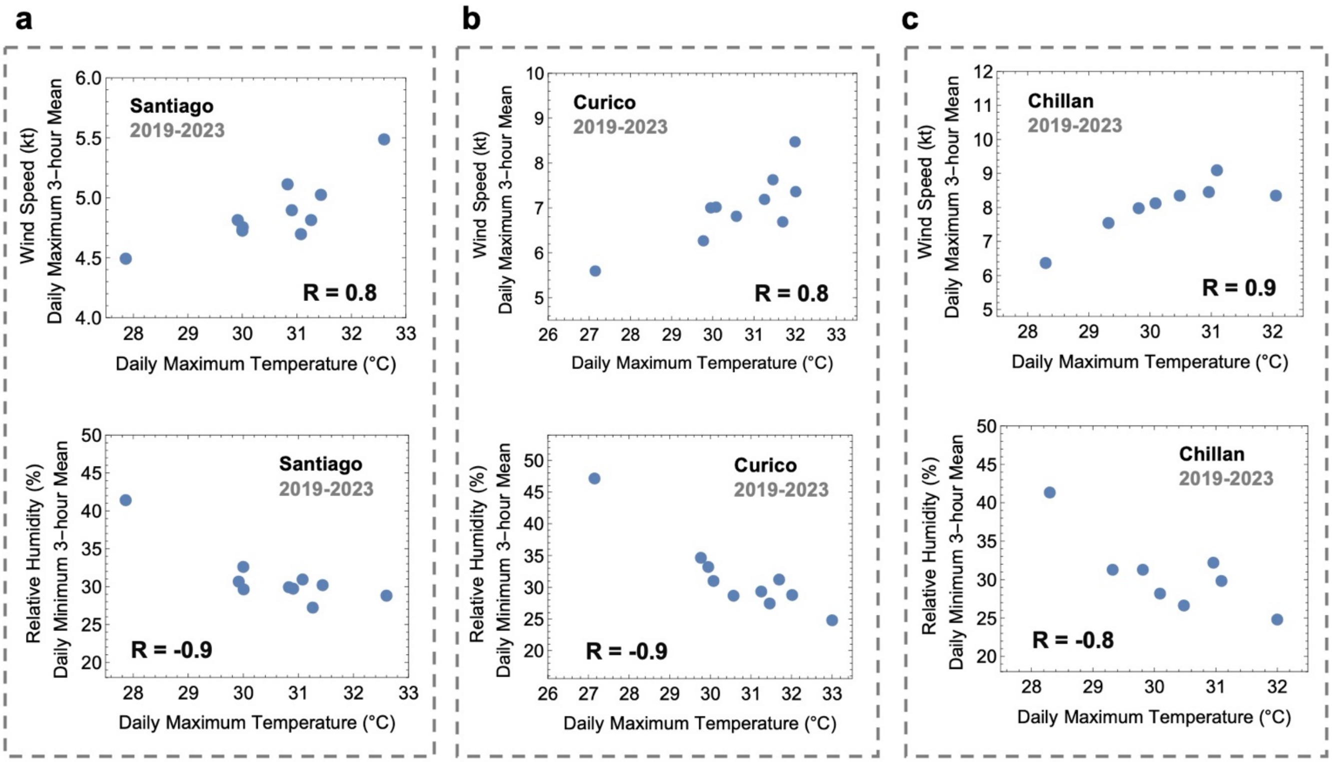


**Fig. S3**

Scatter plots between monthly means (January and February) of the daily maximum temperatures the concurrent monthly means of the daily maximum wind speed (first row), and the concurrent monthly means of the daily minimum relative humidity (second row). The correlation coefficients (R) are shown in the plots.

a) Santiago (33°27'25"S, 70°38'54"W).
b) Curico (34°58'58"S, 71°14'22"W).
c) Chillan (36°36'24"S, 72°6'12"W).

The relative humidity and the wind speed were averaged from 3 to 6 pm local time Weather measurements (daily maximum temperature, wind speed and relative humidity) come from Chilean Weather Service (DMC): https://climatologia.meteochile.gob.cl/application/index/menuTematicoEmas. Plots were generated using Python's Matplotlib library, version 3.4.3, https://matplotlib.org/3.4.3/contents.html.

**“Large” fires (>200 ha.) account for about 70% of the annual burned area in Chile.**

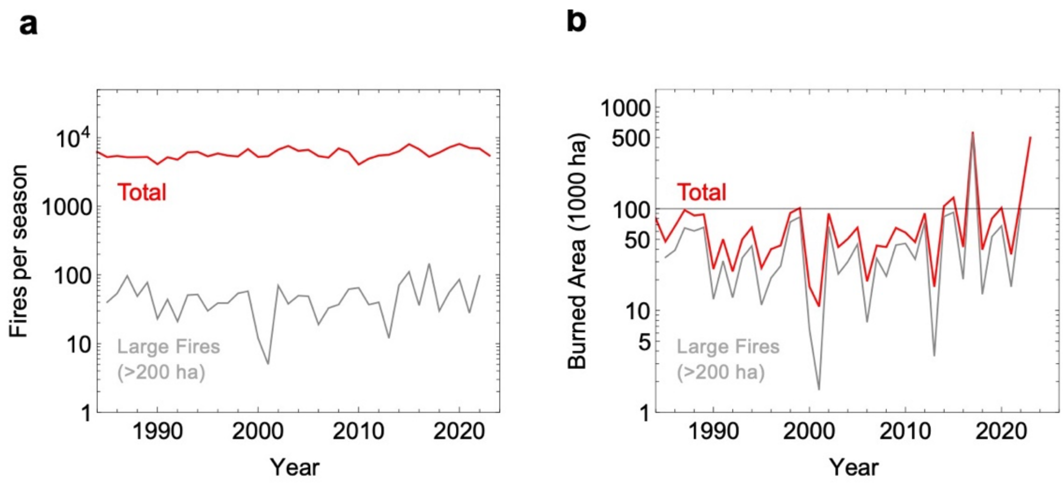


**Fig. S4**

a) Annual number of fires and “large” fires in Chile. “Large” fire is an arbitrary designation, e.g., the Chilean Forestry Agency (CONAF) considers it to be 200 ha. or more[19]. Large fires account for about 1% of the nearly 6,000 fires recorded every year.

b) Area annually burned in Chile. “Large” fires account for about 70% of the annual burned area in the country. Ranked by the annual burned area, six of the seven most destructive fire seasons on record occurred since 2014, and a total of about 1.7 million ha. burned during the last decade in the country.

Wildfire data comes from the Chilean Forestry Agency (CONAF) available at https://www.conaf.cl/incendios-forestales/incendios-forestales-en-chile/estadisticas-historicas/. Plots were generated using Python’s Matplotlib library, version 3.4.3, https://matplotlib.org/3.4.3/contents.html.

**Heatwaves have surged during the last decade in Central Chile.**

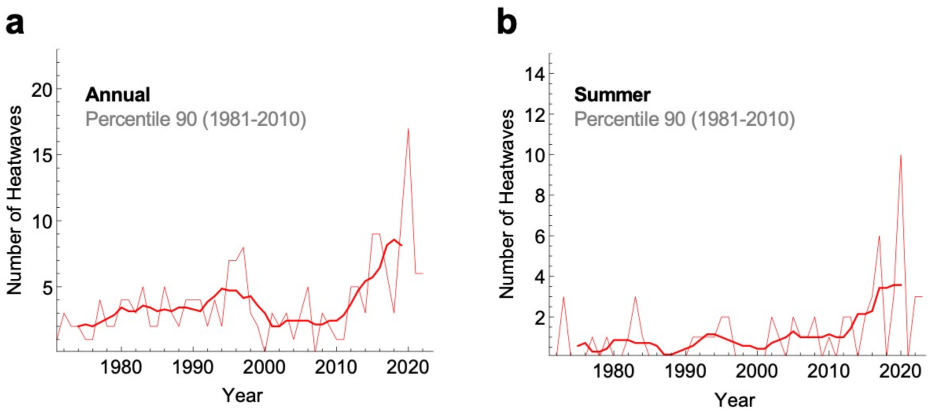


**Fig. S5**

Progress of heatwaves in our study area. Bold lines show 7-year centered moving averages.

a) Number of heatwaves per year.
b) Number of heatwaves per summer.

A heatwave is a period of at least 3 consecutive "very warm" days. A day is considered to be "very warm" if the corresponding maximum temperature falls above the 90th percentile of the daily base climatology (built up by using daily maximum temperatures measured over a 30-year base period 1981–2010; see "Methods"). The daily maximum temperature was computed averaging ERA5 reanalysis data[34] across our study area. ERA5 reanalysis data are available at https://www.ecmwf.int/en/forecasts/datasets/reanalysis-datasets/era5. Plots were generated using Python's Matplotlib library, version 3.4.3, https://matplotlib.org/3.4.3/contents.html.

**Extreme Fire Weather conditions have frequently occurred in recent years.**

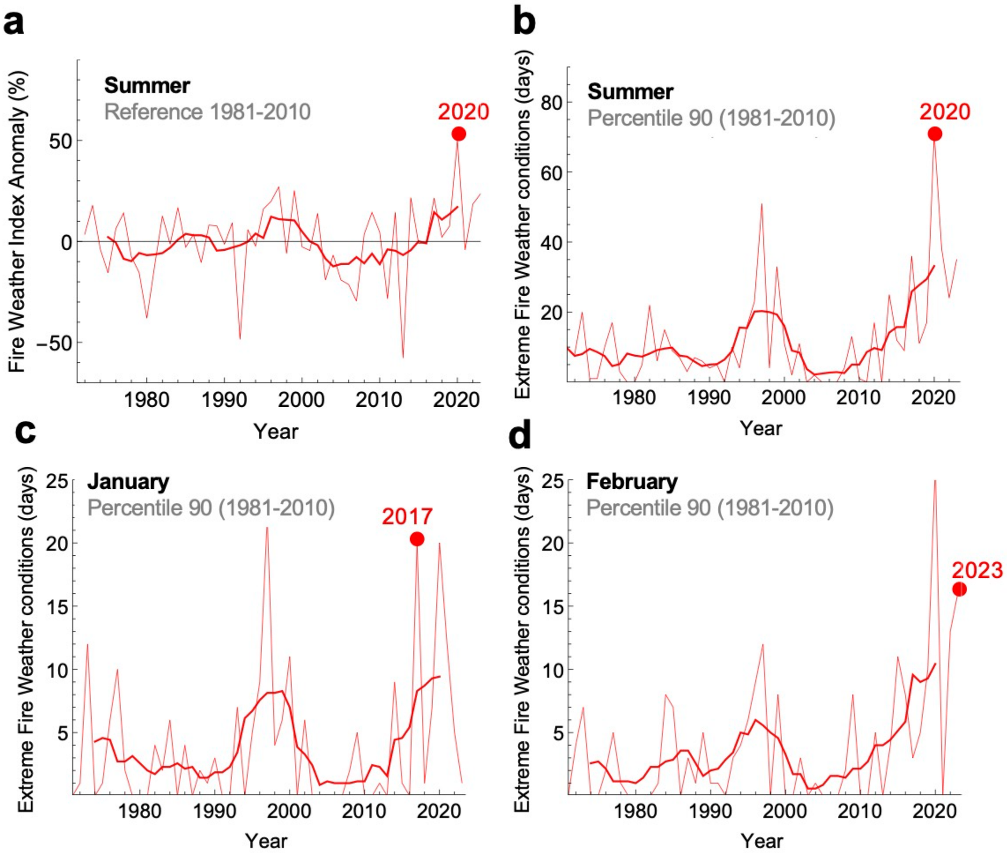


**Fig. S6**

Progress of the Fire Weather Index (FWI) averaged across our study area. Bold red lines show 5-year centered moving averages.

a) Summer (December-January-February) FWI anomalies relative to the 1981-2010 mean. Five of the ten largest positive FWI anomalies occurred since 2014.
b) Number of Summer (December-January-February) extreme fire weather days. Ranked by frequency of extreme fire weather conditions, six of the ten worst seasons occurred since 2014.
c) Number of extreme fire weather days (January).
d) Number of extreme fire weather days (February).

Fire weather conditions were considered to be "extreme" if the corresponding fire weather index falls above the 90th percentile of the daily base climatology (built up by using daily FWI values computed over a 30-year base period 1981–2010; see "Methods"). Daily values of the FWI were computed using estimates of the precipitations, near-surface wind speed, near-surface temperature, and the relative humidity from the ERA5 reanalysis data available at https://www.ecmwf.int/en/forecasts/datasets/reanalysis-datasets/era5. Plots were generated using Python's Matplotlib library, version 3.4.3, https://matplotlib.org/3.4.3/contents.html.

**The tropical Pacific surface temperature (especially in the Niño 1+2 region) is particularly important for Central Chile.**

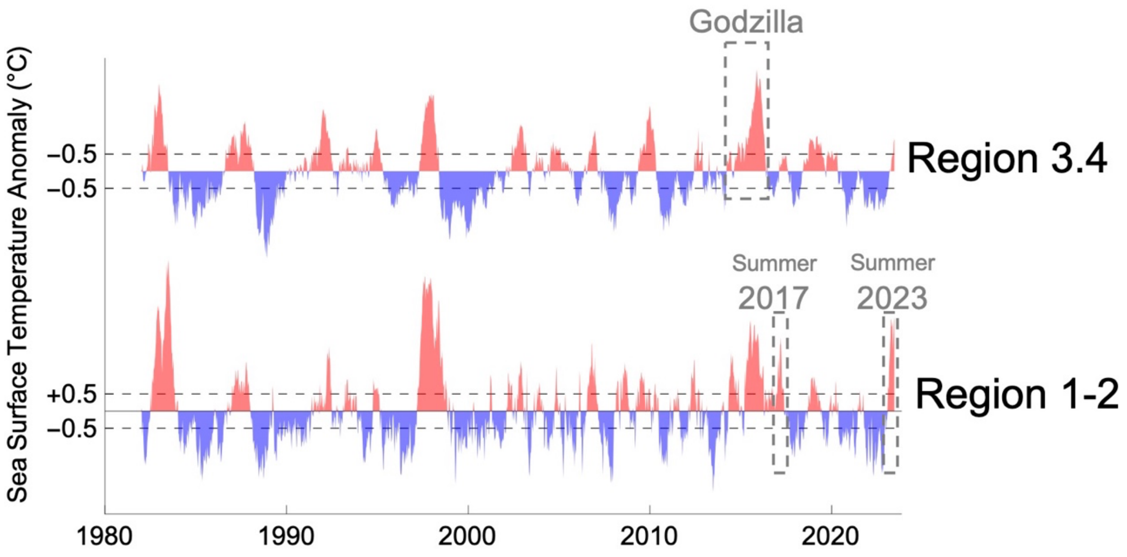


**Fig. S7**

Weekly anomalies of the sea surface temperature (SST) in two important Niño regions. The fierce fires in summer 2017 and summer 2023 in Central Chile concurred with positive anomalies in the Niño 1+2 region (see dotted rectangular boxes in the lower plot).

The dotted rectangular box in the upper plot highlights the 2015 El Niño event, that due to its record intensity in the Niño 3.4 region is often referred to as El Niño Godzilla. While intense, the 2015 El Niño event did not exhibit record positive anomalies in the Niño 1+2 region (like in 1982 and 1997).

Weekly SST anomalies come from the Climate Prediction Center (CPC), part of the National Oceanic and Atmospheric Administration (NOAA) available at https://www.cpc.ncep.noaa.gov/data/indices/wksst8110.for. Plots were generated using Python's Matplotlib library, version 3.4.3, https://matplotlib.org/3.4.3/contents.html.

**El Niño makes summer generally warmer in Central Chile**

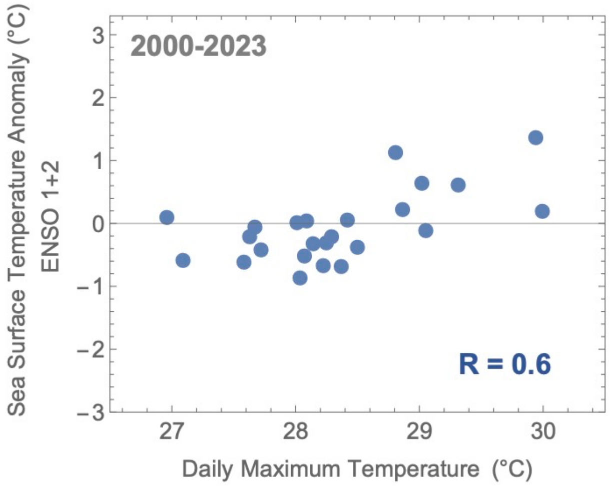


**Fig. S8**

Scatter plot of the sea surface temperature (SST) in the Nino 1+2 region and the daily maximum temperature averaged across our study area. The correlation coefficient is shown in the plot.

SST anomalies (averaged from January to March) come from the Climate Prediction Center (CPC), part of the National Oceanic and Atmospheric Administration (NOAA), available at https://www.cpc.ncep.noaa.gov/data/indices/wksst8110.for. The daily maximum temperature (averaged for January-February, at the height of the southern hemisphere's summer) comes from ERA5 reanalysis data available at https://www.ecmwf.int/en/forecasts/datasets/reanalysis-datasets/era5. Plots were generated using Python's Matplotlib library, version 3.4.3, https://matplotlib.org/3.4.3/contents.html.

**The sea surface temperatures in the Niño 1+2 region and in the Chile Niño/Niña Region (a region directly off the coast of northern Chile) appear to be coupled.**

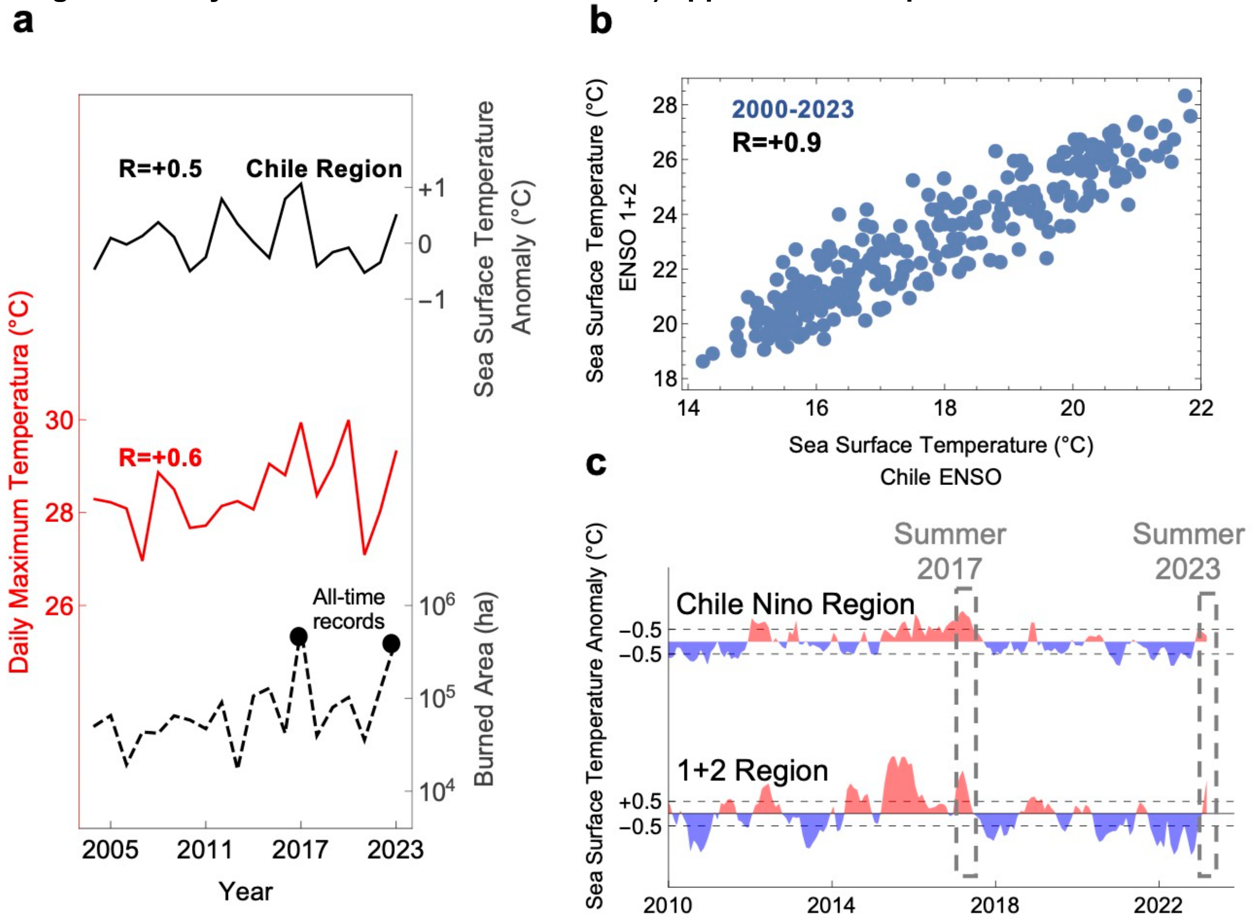


**Fig. S9**

a) Area annually burned in Central Chile (black dotted line), daily maximum temperature (red line) averaged across Central Chile, and sea surface temperature (SST) anomalies in the Chile Niño / Niña region (black line). There is a relatively high correlation between the burned area and the daily maximum temperature (R=+0.6). The correlation is slightly lower (R=+0.5) between the burned area and the SST anomaly in the Chile Niño / Niña region.

b) Scatter plot of the monthly sea surface temperatures (SST) in the Niño 1+2 region (right in front of the western coast of Peru; 0–10°S, 90°W–80°W) and in the Chile Niño/Niña region (directly off the coast of northern Chile; 20°-34°S, 70°W–80°W). The correlation coefficient (R=+0.9) is shown in the plot.

c) Monthly SST anomalies in the Niño 1+2 region and in the Chile Niño/Niña region. The fierce fires in summer 2017 and summer 2023 in Central Chile concurred with positive anomalies in both regions (see dotted rectangular boxes in the plots).

Burned area data come from the Chilean Forestry Agency (CONAF)[19] available at https://www.conaf.cl/incendios-forestales/incendios-forestales-en-chile/estadisticas-historicas/. SST anomalies (averaged from January to March) in the case of (a) come from the ERA5 reanalysis available at https://www.ecmwf.int/en/forecasts/datasets/reanalysis-datasets/era5. Daily maximum temperature (averaged for January-February, at the height of the southern hemisphere's summer) also come from the ERA5 reanalysis. Plots were generated using Python's Matplotlib library, version 3.4.3, https://matplotlib.org/3.4.3/contents.html.

**Temperatures in the Niño regions decoupled in late 2022 and exhibited remarkable contrasting anomalies in late summer 2023**

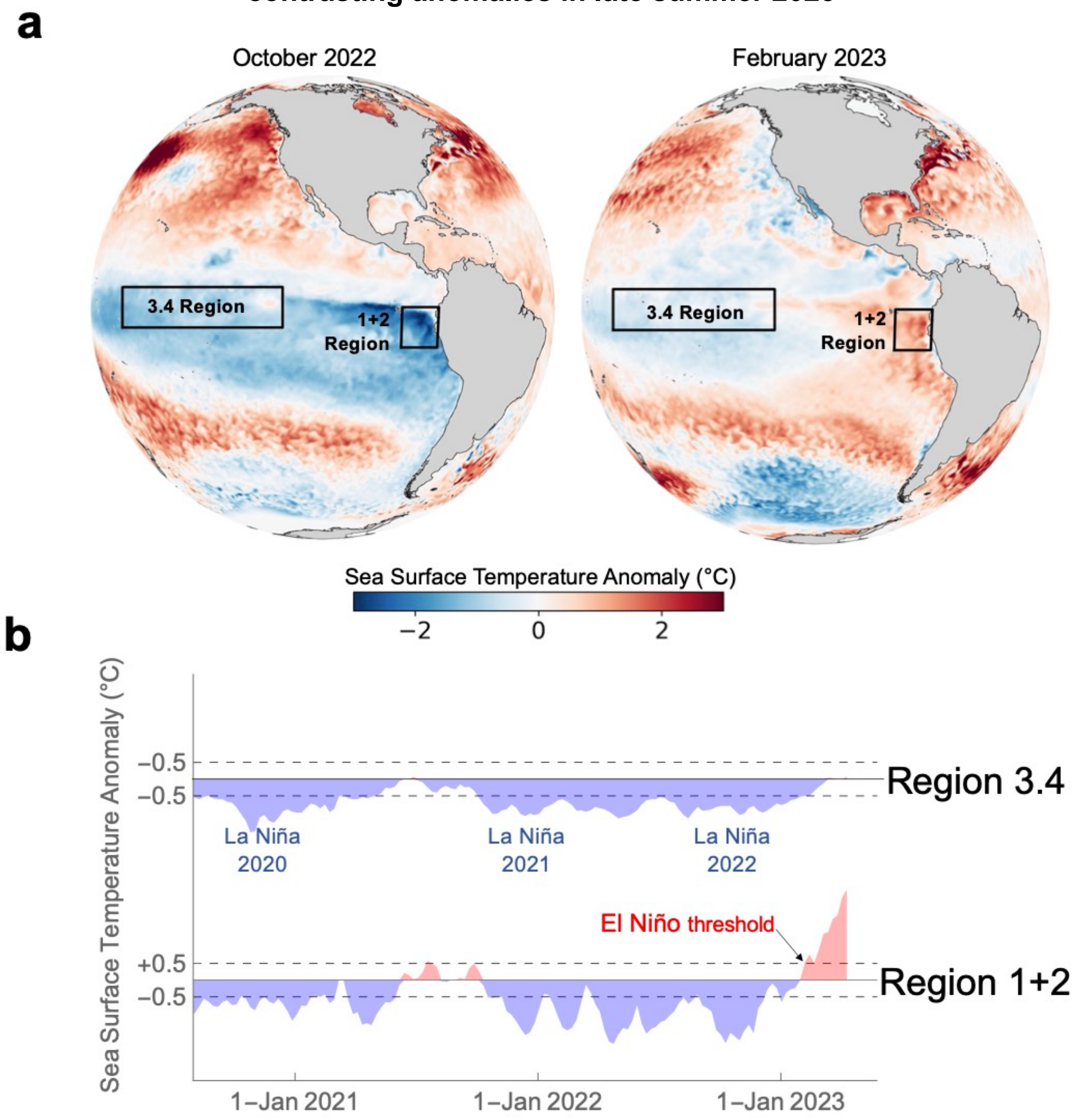


**Fig. S10**

a) While negative anomalies in the sea surface temperature (SST) prevailed in all the Niño regions in late 2022 (i.e., October 2022, left side plot), contrasting anomalies were apparent in late summer 2023 (i.e., February 2023, right side plot). In February 2023, negative anomalies (La Niña) persisted in the Niño 3.4 region whilst positive anomalies (La Niña) prevailed in the Niño 1+2 region.

b) Weekly SST anomalies in the Niño regions. A triple-dip La Niña occurred from 2020 to 2022 in both the Niño 3.4 region and the Niño 1+2 region. However, temperatures in the Niño 3.4 region and in the Niño 1+2 region decoupled in late 2022. In early December 2022, the SST anomaly in the Niño 1+2 region crossed the -0.5°C threshold, ending *de facto* La Niña in the Niño 1+2 region.

In the case of (a), SST anomalies come from the ERA5 dataset produced by the European Centre for Medium-range Weather Forecasts (ECMWF). In the case of (b), weekly SST anomalies come from the Climate Prediction Center (CPC), part of the National Oceanic and Atmospheric Administration (NOAA), available at https://www.cpc.ncep.noaa.gov/data/indices/wksst8110.for. Plots were generated using Python's Matplotlib library, version 3.4.3, https://matplotlib.org/3.4.3/contents.html.

**The Southern Annular Mode (SAM) index appears to be becoming increasingly positive.**

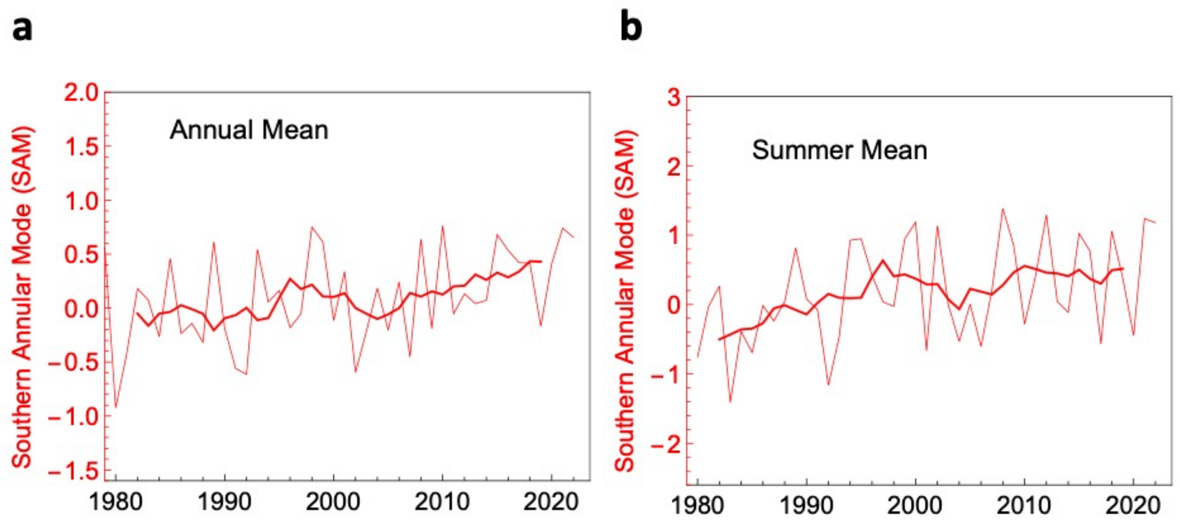


**Fig. S11**

a) Annual mean of the Southern Annular Mode (SAM) index. There is a long-term trend toward positive values of the SAM index. In 2022, the annual mean of the SAM index ended in positive values for the third year in a row.

b) Mean SAM index for summer (December-January-February, DJF). Attributable to the success of the Montreal Protocol, there has been a pause in the long-term strengthening of the summertime SAM.

Bold lines show 7-year centered moving averages. Estimates of the SAM index were obtained from Climate Prediction Center (National Weather Service, National Oceanic and Atmospheric Administration – NOAA). Daily estimates of the SAM index are available at: https://www.cpc.ncep.noaa.gov/products/precip/CWlink/daily_ao_index/aao/aao.shtml#publication. Plots were generated using Python's Matplotlib library, version 3.4.3, https://matplotlib.org/3.4.3/contents.html.

**Cost of fighting wildfires rocketed during the last decade.**

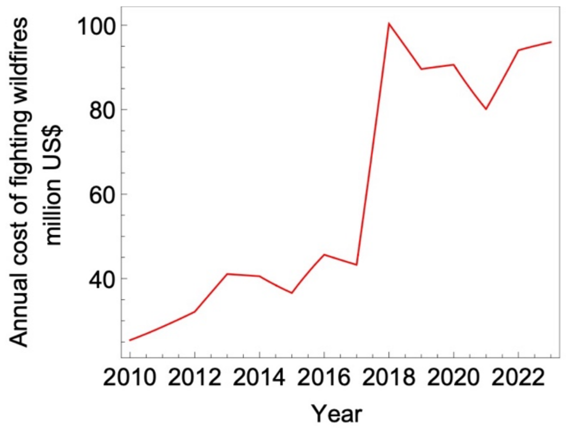


**Fig. S12**

Annual resources (in 2022 U.S. dollars) for fighting wildfires allocated by the Chilean government during the last decade. The last fire season alone, the Chilean government and the forest industry allocated a total of about 180 million dollars for fighting fires. Plots were generated using Python's Matplotlib library, version 3.4.3, https://matplotlib.org/3.4.3/contents.html.

**Plantations account for about 18% of the Chilean forests and for about 30% of the burned area.**

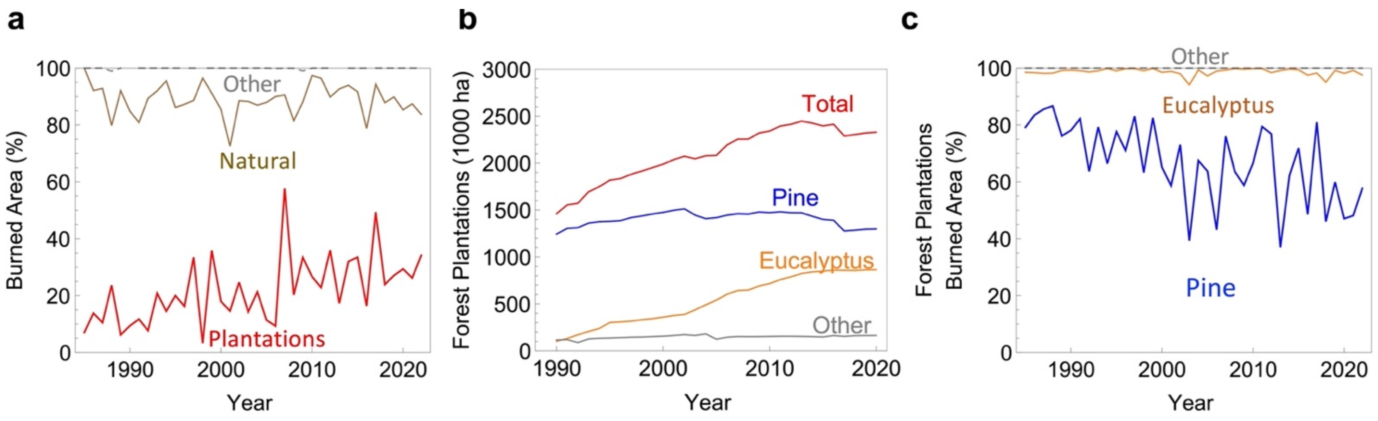


**Fig. S13**

a) Share of burned area by land cover class. The relative weight of plantation forests in the burned area has nearly double since the early 1990s.

b) Planted forest area in Chile. Four of the Chilean administrative Regions in our study area account for most of the tracts of forest land in the country: Maule (0.6 million ha.), Ñuble (0.3 million ha.), Biobio (0.9 million ha), and Araucania (0.4 million ha.). Eucalyptus plantations have climbed since the early 1990s and now they account for about 40% of the planted forest area in Chile.

c) Share of burned area by plantation type. The relative weight of eucalyptus plantations in the burned area of planted forest has nearly doubled since the early 1990s.

Statistical significance tests confirm that the response (i.e., the burned area) of natural and plantation forests are not significantly different from each other (Tables S4-S5). Fire data come from the Chilean Forestry Agency (CONAF) available at https://www.conaf.cl/incendios-forestales/incendios-forestales-en-chile/estadisticas-historicas/. Forestry statistics come from Chilean Office for Agricultural Studies and Policies (ODEPA) (see https://www.odepa.gob.cl/estadisticas-del-sector/estadisticas-productivas) and from CONAF (see https://www.conaf.cl/nuestros-bosques/bosques-en-chile/catastro-vegetacional). Plots were generated using Python's Matplotlib library, version 3.4.3, https://matplotlib.org/3.4.3/contents.html.

Table S1. Main characteristics of the climate in the Chilean administrative Regions considered in this study (note that climate conditions can vary throughout the Region due to its diverse geography and microclimates). The information is based on Garreaud, R. The Andes climate and weather. Adv. Geosci. 22, 3–11 (2009), and on Harris, I., *et al.* Version 4 of the CRU TS monthly high-resolution gridded multivariate climate dataset. *Sci. Data* **7**, 109 (2020). Additional data are shown in Fig. S1.

| Region | Climate |
|---|---|
| Coquimbo | The climate in the Coquimbo Region is generally characterized as arid and semi-arid. It experiences warm, dry summers with daily maximum temperatures often exceeding 20°C, and mild winters with minimal rainfall. Coastal areas benefit from the moderating influence of the Pacific Ocean, while inland areas can be significantly hotter and drier. The region relies on irrigation for agriculture due to its arid nature. |
| Valparaíso | The climate in the Valparaíso Region of Chile is predominantly Mediterranean, characterized by mild, wet winters and warm, dry summers. Daily maximum temperatures range from 20°C in winter to 25°C in summer (although temperatures can be around 10°C higher in the valleys). The coastal areas enjoy a maritime influence, while further inland, temperatures can be more extreme. Dry summers have led to water shortages in recent years. |
| Metropolitana | The climate in the Metropolitana Region is typically characterized as a Mediterranean climate with distinct seasons. Summers are warm and dry, with daily maximum temperatures ranging from 20 to 25°C although temperatures can be around 10°C higher in the valleys). Most precipitation occurs during the winter months that are mild and wet, with daily maximum temperatures around 10 to 15°C. |
| O'Higgins | The O'Higgins Region features a Mediterranean climate characterized by warm, dry summers and mild, wet winters. Summers typically have daily maximum temperatures around 23-28°C, with little rainfall. Winters are cooler, with daily maximum temperatures around 10-15°C and increased rainfall, which contributes to the region's agricultural productivity. The contrast between the summer and winter seasons, makes the Region suitable for agriculture and winegrowing. |
| Maule | The Maule Region is characterized by a Mediterranean climate. Summers are typically warm and dry, with daily maximum temperatures ranging from 25°C to 28°C. Winters are mild and relatively wet, with temperatures around 10°C to 15°C. Rainfall is concentrated in the winter months, with an annual average of approximately 700 millimeters. Despite occasional frosts and extreme heat events, the Region is suitable for a large variety of crops. |
| Biobío | The climate in the Biobío Region features warm, dry summers and mild/wet winters. The region experiences a clear distinction between seasons, with daily maximum temperatures averaging around 27°C in summer and about 12°C in winter. Rainfall is concentrated during the winter months, with occasional heavy downpours and floodings. The Mediterranean climate supports a variety of crops and forest plantations. |
| Ñuble | The climate in the Ñuble Region is characterized by warm, dry summers and cool, wet winters. Summers typically have daily maximum temperatures reaching around 25-30°C with limited rainfall. In contrast, winters are cooler, with daily maximum temperatures around 10-15°C, and considerable precipitations. |
| Araucanía | The Araucanía Region is characterized by a temperate climate with distinct seasons. Summers are typically warm, with daily maximum temperatures ranging from 23°C to 28°C. Winters are cooler with daily maximum temperatures between 11°C and 14°C. Rainfall is distributed throughout the year, but winters can be especially rainy. The region is known for its rich green landscapes and forests, as well as for the agriculture and forest plantations. |

Table S2. Analysis of Variance (ANOVA) results showing the statistically significantly different regional groupings. Outputs show degrees of freedom (DF), F statistic and associated p value (Prob>F). Grouping 1 = Coquimbo, Valparaíso. Grouping 2 = Metropolitana, O'Higgins. Grouping 3 = Maule, BioBio, Nuble, Araucanía.

| Source | DF | Sum of Squares $10^6$ | Mean Square $10^6$ | F Ratio | p-value |
|---|---|---|---|---|---|
| **Number of Fires:** | | | | | |
| Model | 2 | 27.3 | 13.6 | 33.2 | <0.0001 |
| Error | 67 | 26.6 | 0.41 | | |
| Total | 69 | 54.9 | | | |
| **Burned Area (x1000 ha.):** | | | | | |
| Model | 2 | 0.02 | 0.010 | 4.9 | 0.01 |
| Error | 67 | 0.13 | 0.002 | | |
| Total | 69 | 0.15 | | | |

Table S3. Mean Difference Test (MDT) outputs showing pairwise comparisons between fire metrics (number of fires and burned area) in the three regional groupings. The first two regional groupings are not significantly different from each other, neither in terms of the number of fires nor in terms of burned area. Only group 3 is significantly different from the others. Grouping 1 = Coquimbo, Valparaíso. Grouping 2 = Metropolitana, O'Higgins. Grouping 3 = Maule, BioBio, Nuble, Araucanía.

| Group | Comparison group | Difference | TestStat | p-value |
|---|---|---|---|---|
| **Number of Fires:** | | | | |
| 1 | 2 | 59 | 0.69 | 0.25 |
| 3 | 2 | 1290 | 7.57 | <0.0001 |
| 3 | 1 | 1232 | 6.55 | <0.0001 |
| **Burned Area (x1000 ha.):** | | | | |
| 1 | 2 | -8.5 | -1.50 | 0.07 |
| 3 | 2 | 29.3 | 2.23 | 0.02 |
| 3 | 1 | 37.9 | 3.17 | 0.002 |

Table S4. Analysis of Variance (ANOVA) results showing the statistically significantly different types of forests. Outputs show degrees of freedom (DF), F static and associated p value (Prob>F). Grouping 1 = Natural. Grouping 2 = Plantations. Grouping 3 = Other (unclassified) land.

| Source | DF | Sum of Squares $10^3$ | Mean Square $10^3$ | F Ratio | p-value |
|---|---|---|---|---|---|
| **Burned Area (x1000 ha.):** | | | | | |
| Model | 2 | 15.3 | 7.6 | 1.9 | 0.16 |
| Error | 27 | 107.8 | 4.0 | | |
| Total | 29 | 123.2 | | | |

Table S5. Mean Difference Test (MDT) outputs showing pairwise comparisons between fire metrics (burned area) in groupings formed according to the type of forest. Natural and plantation forests are not significantly different from each other. Grouping 1 = Natural. Grouping 2 = Plantations. Grouping 3 = Other (unclassified) land.

| Group | Comparison group | Difference | TestStat | p-value |
|---|---|---|---|---|
| **Burned Area (x1000 ha.):** | | | | |
| 1 | 2 | 17.1 | 0.5 | 0.31 |
| 3 | 2 | -36.9 | -1.4 | 0.09 |
| 3 | 1 | -54.1 | -2.4 | 0.02 |

Table S6. Correlation coefficients (R) and p-values between the burned area (per type of forest and species) and relevant variables. Data from the period 2000-2022 were analyzed. Correlations considered significant ($p < 0.05$) are highlighted in bold.

| | | Maximum Air Temperature (°C) | | SST ENSO 1+2 (°C) | | SST ENSO 3.4 (°C) | | Fire Weather Index (FWI) | |
|---|---|---|---|---|---|---|---|---|---|
| | | R | p | R | p | R | p | R | p |
| Burned Area (ha.) | *Total Plantations* | **+0.5** | **0.01** | **+0.5** | **0.01** | +0.1 | 0.81 | +0.3 | 0.11 |
| | Pine | **+0.5** | **0.02** | **+0.6** | **0.01** | +0.0 | 0.84 | +0.3 | 0.16 |
| | Eucalyptus | **+0.6** | **0.00** | **+0.5** | **0.02** | +0.1 | 0.72 | **+0.5** | **0.01** |
| | *Total Natural* | **+0.6** | **0.00** | **+0.5** | **0.01** | +0.1 | 0.58 | **+0.4** | **0.04** |
| | Trees | **+0.6** | **0.00** | **+0.5** | **0.01** | +0.1 | 0.59 | **+0.4** | **0.04** |
| | Pasture | **+0.5** | **0.01** | **+0.4** | **0.05** | +0.2 | 0.35 | +0.3 | 0.11 |
| | Bush | **+0.6** | **0.00** | **+0.5** | **0.02** | +0.1 | 0.71 | **+0.4** | **0.04** |
| | *Other Land* | **+0.7** | **0.00** | **+0.5** | **0.03** | +0.1 | 0.60 | **+0.6** | **0.00** |

Table S7. Correlation coefficients (R) and p-values between fire metrics (per Chilean administrative Region) and relevant variables. Data from the period 2000-2022 were analyzed. Correlations considered significant ($p < 0.05$) are highlighted in bold.

.

| | | Maximum Air Temperature (°C) | | SST ENSO 1+2 (°C) | | SST ENSO 3.4 (°C) | | Fire Weather Index (FWI) | |
|---|---|---|---|---|---|---|---|---|---|
| | | R | p | R | p | R | p | R | p |
| Burned Area (ha.) | Central Chile | **+0.6** | **0.00** | **+0.6** | **0.00** | +0.0 | 0.83 | **+0.5** | **0.04** |
| | Fires >200 ha. | **+0.6** | **0.00** | **+0.6** | **0.00** | +0.0 | 0.88 | **+0.4** | **0.04** |
| | Coquimbo | +0.2 | 0.28 | +0.2 | 0.46 | +0.1 | 0.72 | +0.1 | 0.51 |
| | Valparaíso | +0.4 | 0.08 | **+0.4** | **0.05** | +0.2 | 0.47 | +0.2 | 0.40 |
| | Metropolitana | **+0.4** | **0.03** | **+0.5** | **0.02** | +0.1 | 0.79 | +0.3 | 0.16 |
| | O'Higgins | **+0.4** | **0.03** | **+0.5** | **0.01** | +0.1 | 0.68 | +0.2 | 0.36 |
| | Maule | **+0.5** | **0.01** | **+0.6** | **0.00** | +0.0 | 0.88 | +0.3 | 0.16 |
| | BioBio+Nuble | **+0.5** | **0.03** | **+0.4** | **0.03** | +0.0 | 0.98 | **+0.4** | **0.05** |
| | Araucanía | **+0.4** | **0.03** | +0.1 | 0.56 | +0.0 | 0.95 | **+0.5** | **0.01** |
| Number of Fires | Central Chile | +0.4 | 0.08 | +0.0 | 0.96 | +0.2 | 0.29 | +0.3 | 0.21 |
| | Fires >200 ha. | **+0.6** | **0.00** | +0.3 | 0.11 | +0.3 | 0.25 | **+0.6** | **0.00** |
| | Coquimbo | **+0.5** | **0.01** | +0.3 | 0.15 | +0.2 | 0.25 | +0.2 | 0.33 |
| | Valparaíso | +0.0 | 0.86 | +0.1 | 0.72 | +0.1 | 0.68 | -0.3 | 0.12 |
| | Metropolitana | +0.2 | 0.29 | -0.1 | 0.63 | +0.0 | 0.91 | -0.3 | 0.15 |
| | O'Higgins | **+0.4** | **0.03** | +0.1 | 0.58 | +0.1 | 0.81 | **+0.5** | **0.02** |
| | Maule | **+0.5** | **0.01** | +0.2 | 0.33 | +0.2 | 0.30 | **+0.5** | **0.02** |
| | BioBio+Nuble | +0.3 | 0.21 | -0.1 | 0.62 | +0.1 | 0.54 | +0.2 | 0.26 |
| | Araucanía | +0.3 | 0.09 | +0.1 | 0.76 | +0.2 | 0.42 | +0.3 | 0.11 |
